# Nebulised interferon beta-1a (SNG001) in the treatment of viral exacerbations of COPD

**DOI:** 10.1186/s12931-024-02854-7

**Published:** 2024-05-29

**Authors:** Phillip D. Monk, Jody L. Brookes, Victoria J. Tear, Toby N. Batten, Clare Newall, Marcin Mankowski, Michael G. Crooks, Dave Singh, Rekha Chaudhuri, Brian Leaker, Kerry Lunn, Sophie Reynolds, Sarah Dudley, Felicity J. Gabbay, Stephen T. Holgate, Ratko Djukanovic, Thomas MA Wilkinson

**Affiliations:** 1 Synairgen Research Ltd, Southampton, UK; 2 Veramed, Twickenham, London, UK; 3 tranScrip Ltd, Wokingham, UK; 4 Respiratory Research Group, Hull York Medical School, University of Hull, Kingston Upon Hull, Hull, UK; 5 Medicines Evaluation Unit, The University of Manchester, Manchester University NHS Foundation Trust, Manchester, UK; 6 Gartnavel General Hospital, Glasgow, UK; 7 School of Infection and Immunity, University of Glasgow, Glasgow, UK; 8 Respiratory Clinical Trials Ltd, Fitzrovia Hospital, London, UK; 9 NIHR Southampton Biomedical Research Centre, Clinical and Experimental Sciences, University of Southampton, Southampton, UK

**Keywords:** Chronic obstructive pulmonary disease, Symptom flare up, Interferons, Biomarkers

## Abstract

**Background:**

Respiratory viral infections are major drivers of chronic obstructive pulmonary disease (COPD) exacerbations. Interferon-β is naturally produced in response to viral infection, limiting replication. This exploratory study aimed to demonstrate proof-of-mechanism, and evaluate the efficacy and safety of inhaled recombinant interferon-β1a (SNG001) in COPD. Part 1 assessed the effects of SNG001 on induced sputum antiviral interferon-stimulated gene expression, sputum differential cell count, and respiratory function. Part 2 compared SNG001 and placebo on clinical efficacy, sputum and serum biomarkers, and viral clearance.

**Methods:**

In Part 1, patients (*N* = 13) with stable COPD were randomised 4:1 to SNG001 or placebo once-daily for three days. In Part 2, patients (*N* = 109) with worsening symptoms and a positive respiratory viral test were randomised 1:1 to SNG001 or placebo once-daily for 14 days in two Groups: A (no moderate exacerbation); B (moderate COPD exacerbation [i.e., acute worsening of respiratory symptoms treated with antibiotics and/or oral corticosteroids]).

**Results:**

In Part 1, SNG001 upregulated sputum interferon gene expression. In Part 2, there were minimal SNG001–placebo differences in the efficacy endpoints; however, whereas gene expression was initially upregulated by viral infection, then declined on placebo, levels were maintained with SNG001. Furthermore, the proportion of patients with detectable rhinovirus (the most common virus) on Day 7 was lower with SNG001. In Group B, serum C-reactive protein and the proportion of patients with purulent sputum increased with placebo (suggesting bacterial infection), but not with SNG001. The overall adverse event incidence was similar with both treatments.

**Conclusions:**

Overall, SNG001 was well-tolerated in patients with COPD, and upregulated lung antiviral defences to accelerate viral clearance. These findings warrant further investigation in a larger study.

**Trial registration:**

EU clinical trials register (2017-003679-75), 6 October 2017.

**Supplementary Information:**

The online version contains supplementary material available at 10.1186/s12931-024-02854-7.

## Background

Exacerbations of chronic obstructive pulmonary disease (COPD) account for much of the overall burden of COPD despite currently available therapies, and are thought to be a key reason for disease progression (in particular lung function decline) [1]. Respiratory viral infections, such as the common cold and influenza, are a major driver of these exacerbations [2–4], with growing evidence that viral infections increase susceptibility to subsequent bacterial infection [5, 6]. Thus, there is a strong rationale for the development of antiviral treatments to reduce the impact of viral COPD exacerbations.

Interferon-β is a naturally-occurring protein produced as an immediate local response to viral infection, and that results in antiviral protein production thereby limiting viral replication [7–9]. Importantly, deficiencies in interferon-β-mediated antiviral responses are associated with poorer outcomes in virus challenge studies in patients with COPD [10, 11]. SNG001 is a unique formulation of recombinant interferon-β1a that contains few excipients and has near-neutral pH, making it appropriate for inhaled administration. It is delivered via nebuliser with the aim of reaching a high local concentration within the lower respiratory tract, and has previously been shown to upregulate antiviral biomarker levels in the lungs of patients with asthma [12, 13]. Use of SNG001 has also been investigated in two studies in patients hospitalised due to Coronavirus Disease 2019 (COVID-19) [14, 15]. In the first, patients receiving SNG001 were more likely to improve, and recovered more rapidly, than those receiving placebo [14]. The second suggested that SNG001 may prevent progression to severe disease, although the primary endpoint was not met [15]. Other interferon formulations (typically given by injection) have been used to treat chronic hepatitis, although with limited efficacy [16].

The efficacy of SNG001 had not previously been evaluated in COPD. The aims of this exploratory, two-part study, the first in COPD, were to provide evidence of proof-of-mechanism (in terms of upregulation of antiviral defences), to demonstrate promotion of viral clearance, and to evaluate the efficacy, tolerability and safety of SNG001 in patients with COPD. Given this was a new population for SNG001, Part 1 was designed as a conventional clinical pharmacology proof-of-concept study. Key results from Part 1 were presented at the 2019 European Respiratory Society annual conference [17].

## Methods

This was a two-part, double-blind, placebo-controlled, exploratory study conducted in non-hospitalised patients.

### Objectives

Given the exploratory nature of the study, none of the objectives were prespecified as primary or secondary, and there was no hierarchy applied to the endpoints.

### Part 1

To assess the safety and tolerability of SNG001, and its effects on antiviral interferon-stimulated gene [ISG] expression in induced sputum, sputum differential cell count, and respiratory function (forced expiratory volume in 1 s [FEV_1_], and peak expiratory flow [PEF]) in patients with stable COPD.

### Part 2

To compare SNG001 versus placebo in terms of efficacy and sputum and serum biomarkers (including ISGs), and to evaluate safety and tolerability in patients experiencing an acute viral infection. Efficacy endpoints included lung function, sputum viral load, Breathlessness, Cough and Sputum Scale (BCSS), COPD Assessment Test (CAT) total score, and reliever medication use.

### Design

In Part 1, patients were randomised 4:1 to SNG001 6 MIU or placebo once-daily for three days (see supplement), and were followed until four days after the last dose. Lung function was assessed pre- and up to 8 h post-dose on Day 1, pre- and up to 1 h post-dose on Days 2 and 3, and on Day 4, with sputum induced at screening, and pre-dose on Days 2 and 4 (see Additional file 1).

In Part 2, patients entered a pre-treatment observation phase, progressing to the treatment phase only if they developed upper respiratory symptoms and/or their COPD symptoms worsened, and they then tested positive for a respiratory virus (BioFire Respiratory panel, bioMerieux, Marcy-l’Étoile, France). During the pre-treatment phase, patients received a daily text message asking about the onset of cold symptoms and/or a deterioration in their COPD symptoms. As soon as this was detected, patients were asked to either respond to the text message or telephone the site, so that study medication could be initiated within 48 h of the onset of respiratory virus symptoms and/or deterioration in COPD symptoms if the treatment phase eligibility criteria were met. Patients were randomised 1:1 to SNG001 6 MIU or placebo once-daily for 14 days, stratified into two Groups: A (cold symptoms and/or COPD symptoms deterioration without moderate COPD exacerbation) and B (moderate COPD exacerbation with or without cold symptoms). Moderate exacerbations were defined as acute worsening of respiratory symptoms treated with short-acting bronchodilators plus antibiotics and/or oral corticosteroids (the choice of which was left to the investigators). Pre-dose on Day 1 (baseline) and post-dose on Days 4, 7, 10 and 13 (treatment phase), and 17 and 28 (follow-up), CAT, FEV_1_ and forced vital capacity (FVC) were assessed, with blood sampled for biomarkers. Sputum was induced at selected sites if a spontaneous sample could not be produced (supplement), but sputum cultures were not performed. Patients recorded BCSS, PEF and reliever medication use daily.

Both parts recruited patients diagnosed with COPD ≥ 12 months prior to entry. The main Part 1 inclusion criteria were post-bronchodilator FEV_1_ ≥ 40% predicted at screening and ≥ 30% prior to the first dose, with sputum production on most days. The main pre-treatment Part 2 inclusion criteria were post-bronchodilator FEV_1_ ≥ 30% predicted (≥ 40% for the first 16 patients), ≥ 1 exacerbation requiring oral corticosteroids/antibiotics in the prior year, and COPD previously worsened by a respiratory virus, with patients excluded if they had a severe exacerbation. Patients provided written informed consent prior to any study-related procedure. See Additional file 1 for full inclusion/exclusion criteria.

The study was approved by a central independent ethics committee, performed in accordance with the Declaration of Helsinki and Good Clinical Practice, and registered at the EU clinical trials register (2017-003679-75).

The protocol was amended nine times. Most of the changes were minor, and were to clarify existing wording. The main amendment followed a review of safety data by the Data Safety Monitoring Committee for the first 16 patients after they had completed treatment in Part 1. This resulted in the post-bronchodilator FEV_1_ inclusion criterion for Part 2 being changed from 40 to 30% predicted. In addition, the sample size for Part 2 was re-estimated. This was originally based on gene expression data, and required 40 patients to be recruited per treatment, which was considered also sufficient to evaluate the clinical endpoints, including BCSS.

### Interventions

In both parts, patients were randomised to treatment according to a pre-specified randomisation schedule. In Part 2, study medication was supplied in blocks of four consecutive randomisation numbers per Group, with randomisation numbers allocated sequentially at each site within each Group. The first dose of study medication was to be administered no later than 48 h after the onset of (or deterioration in) symptoms, or the start of the moderate COPD exacerbation.

Study medication was administered double-blind in both parts of the study, with patients and investigators blinded to treatment assignment using matching placebo. SNG001 was administered at a dose of 6 MIU (in a volume of 0.5 mL) via portable mesh nebuliser (I-neb, Philips Respironics). In Part 1, medication was administered at the study site for all doses; in Part 2 the first dose was administered at the study site, with subsequent doses taken either at the study site or in the patients home.

### Sample size and statistical methods

Part 1 was not formally powered; 10 patients (eight receiving SNG001 and two placebo) was considered sufficient based upon previous findings in patients with asthma [13]. For Part 2, 55 patients per arm were estimated to detect a BCSS SNG001–placebo difference of ± 1.2 with probability (power) of 0.8 [18, 19]. To allow for missing data, 60 patients per arm were required.

For Part 1, changes from baseline in biomarkers were analysed for SNG001 using a mixed model for repeated measures (MMRM), with observed values as the dependent variable, baseline as covariate and visit as fixed effect. Other endpoints were evaluated using summary statistics only.

All results in Part 2 are presented separately for Group A and Group B. The change from baseline in BCSS daily score was analysed using a MMRM, including terms for treatment, Group, smoking status, baseline BCSS score, day, and baseline-by-day and treatment-by-day interactions. The model was fitted with an unstructured variance-covariance matrix, where possible. A similar model was used for the analysis of clinic spirometry, CAT score, home PEF and reliever medication data. There were no adjustments for multiplicity.

Exploratory analyses of sputum antiviral-stimulated genes, viral load clearance and sputum purulence were conducted following unblinding of the study data. Sputum antiviral-stimulated genes were analysed over time using a MMRM with treatment, visit and the treatment by visit interaction as covariates. The models were fitted with an unstructured variance-covariance matrix. Analyses were conducted separately for Group A and Group B.

Viral load clearance for patients with detectable rhinovirus was analysed at each post-baseline visit using a Firth logistic regression model with covariates for treatment and baseline viral load. Viral load clearance was defined as undetectable rhinovirus viral load. Sputum purulence, defined as sputum colour classified as 3, 4 or 5 according to the BronkoTest colour chart was analysed at each visit using Fisher’s exact test. Analyses were conducted separately for Group A and Group B.

The proportion of patients whose symptoms had returned to normal were compared between treatments using a generalised linear mixed model with a logit link function, including terms for treatment, Group, smoking status, visit and treatment by visit interactions. The sputum sample data were summarised by treatment and Group, with data from spontaneous and induced samples combined. In both parts of the study the intention-to-treat (ITT) population was all randomised patients who received at least one dose of study medication, and was used for all efficacy analyses. The safety population, used for all safety analyses, was the same as the ITT population.

## Results

### Participants

**Fig. 1:**
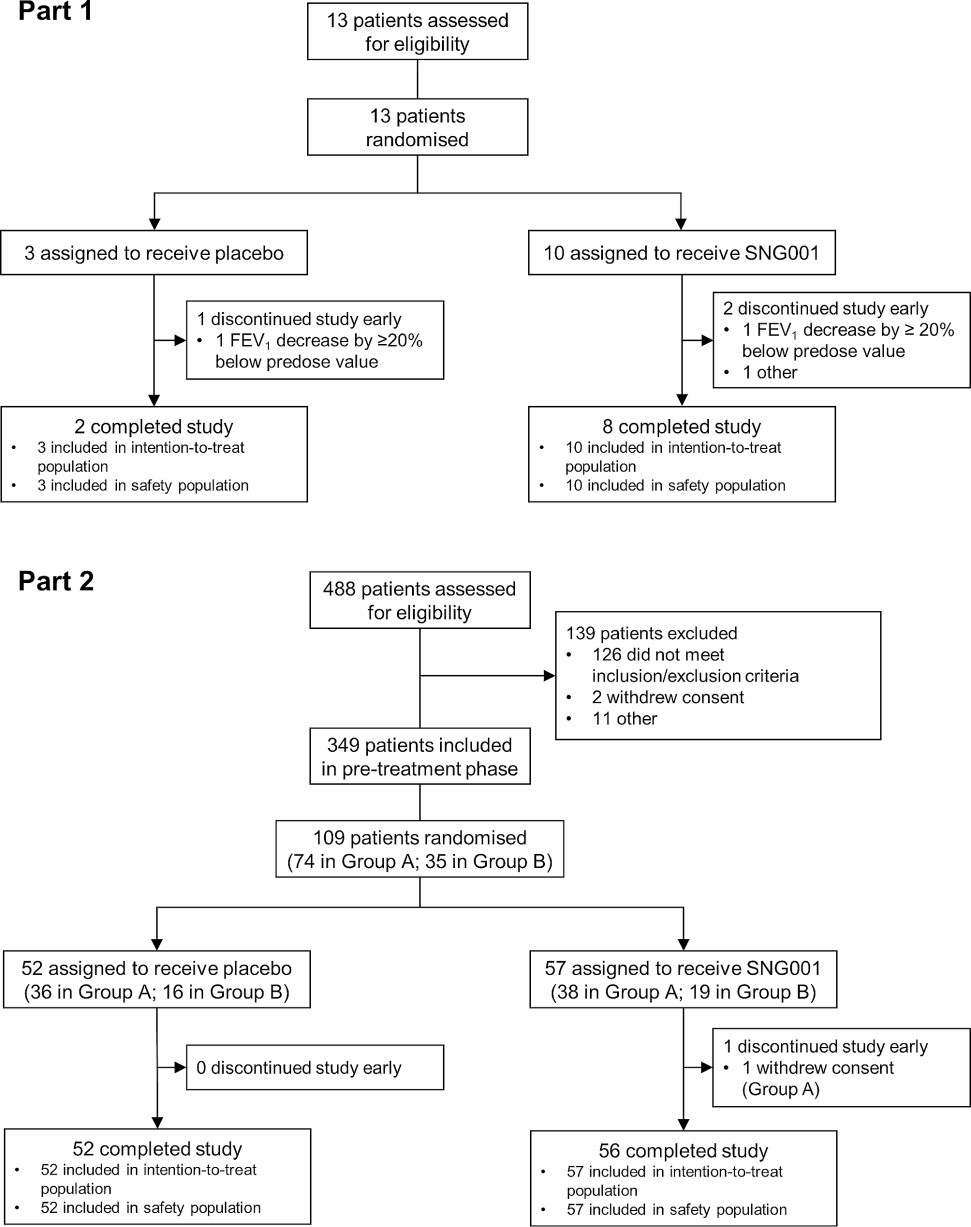
Patient flow through the two parts of the study Group **A**: Patients with cold-like symptoms and/or a deterioration in COPD symptoms, but without a moderate COPD exacerbation. Group **B**: Patients with a moderate COPD exacerbation with or without cold symptoms. FEV_1_, forced expiratory volume in 1 s; COPD, chronic obstructive pulmonary disease

Part 1 was conducted between 5 February and 7 June 2018 at a single UK site, with Part 2 between 3 December 2018 and 5 May 2020 at 16 UK sites. Recruitment into Part 2 was terminated early due to the COVID-19 pandemic. Of the 13 patients randomised into Part 1, 10 completed the study (Fig. 1). Two of the patients who withdrew had an FEV_1_ decrease from baseline ≥ 20% (one in each treatment arm), a pre-specified stopping rule (supplement); neither were symptomatic. The baseline demographics and disease characteristics were consistent across the two treatment arms (Table 1). In Part 2, a total of 351 patients entered the pre-treatment phase, with 109 subsequently meeting the treatment phase inclusion criteria, 108 of whom completed the study (Fig. 1). The baseline demographics and disease characteristics were generally consistent across the two treatments within each Group (Table 1).

**Table 1:** Patient baseline demographics and disease characteristics

	Part 1		Part 2			
			Group A (cold-like symptoms and/or deterioration in COPD symptoms)		Group B (moderate COPD exacerbation)	
	Placebo (*N* = 3)	SNG001 (*N* = 10)	Placebo (*N* = 36)	SNG001 (*N* = 38)	Placebo (*N* = 16)	SNG001 (*N* = 19)
Age, years	67.7 (NC)	67.1 (5.13)	64.6 (6.52)	66.9 (7.14)	66.8 (8.75)	67.2 (7.71)
Sex, male	2 (66.7%)	7 (70.0%)	22 (61.1%)	24 (63.2%)	9 (56.3%)	10 (52.6%)
BMI, kg/m^2^	26.0 (NC)	29.5 (6.01)	28.7 (5.98)	29.0 (6.39)	28.5 (5.12)	28.4 (6.24)
Race						
White	3 (100%)	10 (100%)	33 (91.7%)	35 (92.1%)	16 (100%)	19 (100%)
Smoking status						
Current smoker	2 (66.7%)	1 (10.0%)	17 (47.2%)	15 (39.5%)	4 (25.0%)	6 (31.6%)
Former smoker	1 (33.3%)	9 (90.0%)	19 (52.8%)	23 (60.5%)	12 (75.0%)	13 (68.4%)
Smoking pack-years	40.3 (NC)	38.1 (14.39)	43.0 (23.49)	51.0 (30.36)	48.8 (17.73)	52.2 (20.28)
CAT total score	22.0 (NC)	19.7 (5.12)	19.8 (8.38)	20.4 (6.28)	21.0 (8.88)	24.3 (6.31)
Post-bronchodilator FEV_1_						
L	1.83 (NC)	1.74 (0.394)	1.71 (0.568)	1.64 (0.549)	1.56 (0.348)	1.63 (0.599)
% predicted	70.3 (NC)	62.0 (11.11)	58.4 (13.74)	59.6 (15.05)	58.1 (16.13)	61.7 (19.46)
Post-bronchodilator PEF, L/min	352.0 (NC)	355.3 (78.74)	329.8 (113.65)	310.4 (108.81)	295.7 (64.42)	301.9 (120.26)
Exacerbations in previous 12 months						
0	1 (33.3%)	5 (50.0%)	0	0	0	0
1	1 (33.3%)	4 (40.0%)	18 (50.0%)	14 (36.8%)	7 (43.8%)	6 (31.6%)
≥ 2	1 (33.3%)	1 (10.0%)	18 (50.0%)	24 (63.2%)	9 (56.3%)	13 (68.4%)
BCSS total score	–	–	5.8 (3.02)	6.8 (2.39)	7.9 (2.77)	7.9 (2.46)
Concurrent COPD medication						
Long-acting muscarinic antagonists	3 (100%)	8 (80.0%)	33 (91.7%)	29 (76.3%)	15 (93.8%)	17 (89.5%)
Long-acting β_2_-agonists	3 (100%)	9 (90.0%)	32 (88.9%)	35 (92.1%)	14 (87.5%)	19 (100%)
Inhaled corticosteroids	3 (100%)	8 (80.0%)	28 (77.8%)	31 (81.6%)	14 (87.5%)	16 (84.2%)

Data are mean (SD) or n (%). COPD, chronic obstructive pulmonary disease; NC, not calculated (note that SDs were not calculated when data were available for ≤ 3 patients); BMI, body-mass index; CAT, COPD Assessment Test; FEV_1_, forced expiratory volume in 1 s; PEF, peak expiratory flow; BCSS, Breathlessness, Cough and Sputum Scale

### Part 1

#### Sputum interferon-stimulated antiviral gene expression

**Fig. 2:**
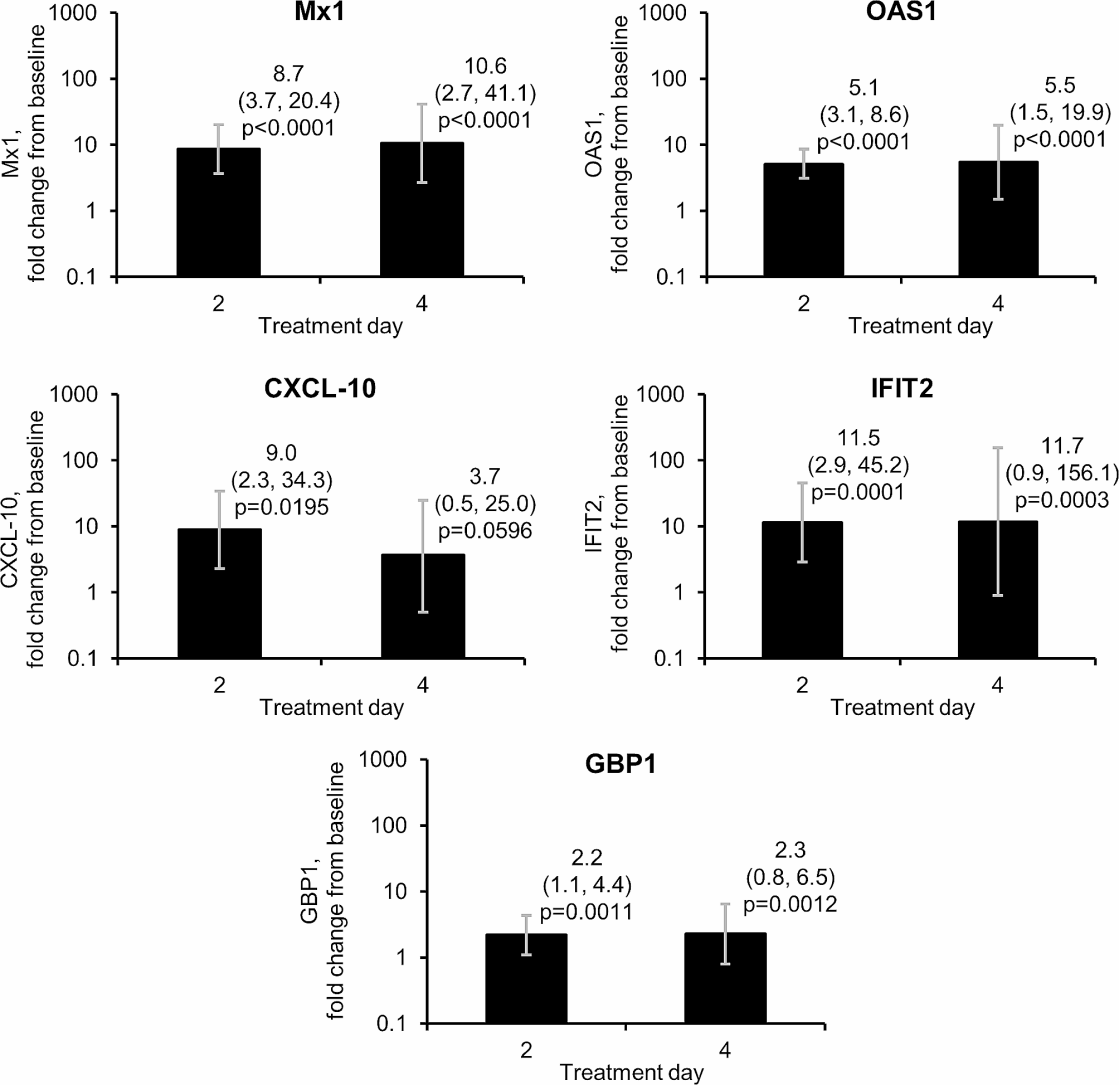
Part 1: Antiviral interferon-stimulated sputum gene expression following administration of SNG001 Data are geometric mean fold change from baseline and 95% confidence intervals. Data available from 6 and 5 patients on Days 2 and 4, respectively, for Mx1, CXCL10, and GBP1, and for 5 and 4 patients, respectively, for OAS1 and IFIT2. Baseline mean (minimum, maximum) values were 3.96 (2.33, 5.28), 2.68 (2.05, 2.99), 1.77 (0.42, 2.90), − 0.20 (–0.87, 0.75) and − 0.24 (–0.57, − 0.05) for Mx1, OAS1, CXCL10, IFIT2 and GBP1, respectively. Values were not calculated for placebo, given data were available for ≤ 3 patients. Mx1, MX dynamin-like GTPase-1; OAS1, 2’-5’-oligoadenylate synthetase; CXCL10, C-X-C motif chemokine ligand-10; IFIT2, interferon-induced protein with tetratricopeptide repeats-2; GBP1, guanylate-binding protein 1

On Day 2 of the treatment period, i.e., approximately 24 h after administration of the first dose, all five assessed ISGs were upregulated from baseline in the SNG001 arm, with between 2.2 and 11.5 fold-changes (Fig. 2, with absolute data in Additional file 1: Table S1). Similar upregulation was also observed on Day 4 (i.e., approximately 24 h after administration of the third dose of SNG001), although significance was lost for C-X-C motif chemokine ligand 10 (CXCL10). Note that values were not calculated for placebo, given data were available for ≤ 3 patients.

#### Safety

The overall incidence of adverse events was similar with the two treatments, with none of the events severe, serious, or leading to treatment discontinuation or death (Table 2). The only preferred term to occur in at least two patients with either treatment was cough, in one patient with placebo and two with SNG001, all related to study treatment but mild in severity. In addition, following administration of SNG001, changes from baseline in sputum differential cell counts were all small (Additional file 1: Table S2). Finally, there was a decrease from baseline with both treatments in FEV_1_ and PEF on Day 1 (Additional file 1: Figures S1 and S2). On Days 2 and 3, changes from baseline with SNG001 were relatively small and variable.

**Table 2:** Part 1: Treatment-emergent adverse events, overall and most common preferred terms (≥ 2 patients with either treatment)

Number of patients (%)	Placebo (*N* = 3)	SNG001 (*N* = 10)
Any adverse event	2 (66.7%)	7 (70.0%)
Cough	1 (33.3%)	2 (20.0%)
Any on-treatment adverse event	1 (33.3%)	4 (40.0%)
Any adverse event related to treatment	2 (66.7%)	3 (30.0%)
Cough	1 (33.3%)	2 (20.0%)
Any severe adverse event	0	0
Any serious adverse event	0	0
Any adverse event leading to discontinuation of study treatment or study withdrawal	0	0
Any fatal adverse event	0	0

### Part 2

#### Clinical efficacy

**Fig. 3:**
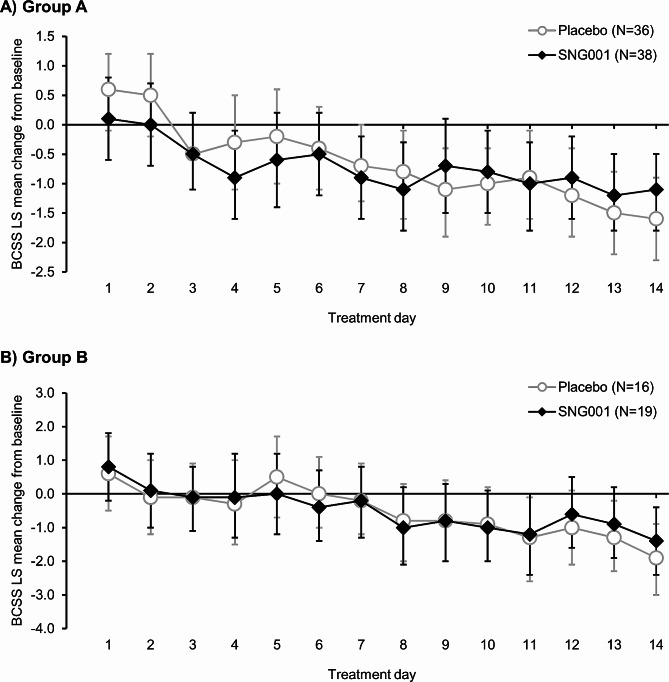
Part 2: BCSS total score change from baseline in (**A**) Group A (cold-like symptoms and/or deterioration in COPD symptoms) and (**B**) Group B (moderate COPD exacerbation) Data are least squares mean and 95% confidence intervals. BCSS, Breathlessness, Cough and Sputum Scale. N values are the intention-to-treat population

There was a gradual decrease from baseline in BCSS total score with both treatments in both Groups, with no statistically significant SNG001–placebo differences (Fig. 3). There were also no SNG001–placebo differences in either CAT total score (Additional file 1: Table S3) or the proportion of patients whose symptoms returned to normal after a moderate exacerbation, although the percentages were consistently higher with SNG001 (Additional file 1: Table S4). Further, reliever medication use was low and did not change from baseline over the treatment period, with no SNG001–placebo differences (least squares mean differences for average use over Days 1–14 of 0.2 [95% CI − 1.2, 1.6] and − 0.4 [–2.4, 1.6] puffs/day in Groups A and B, respectively).

**Fig. 4:**
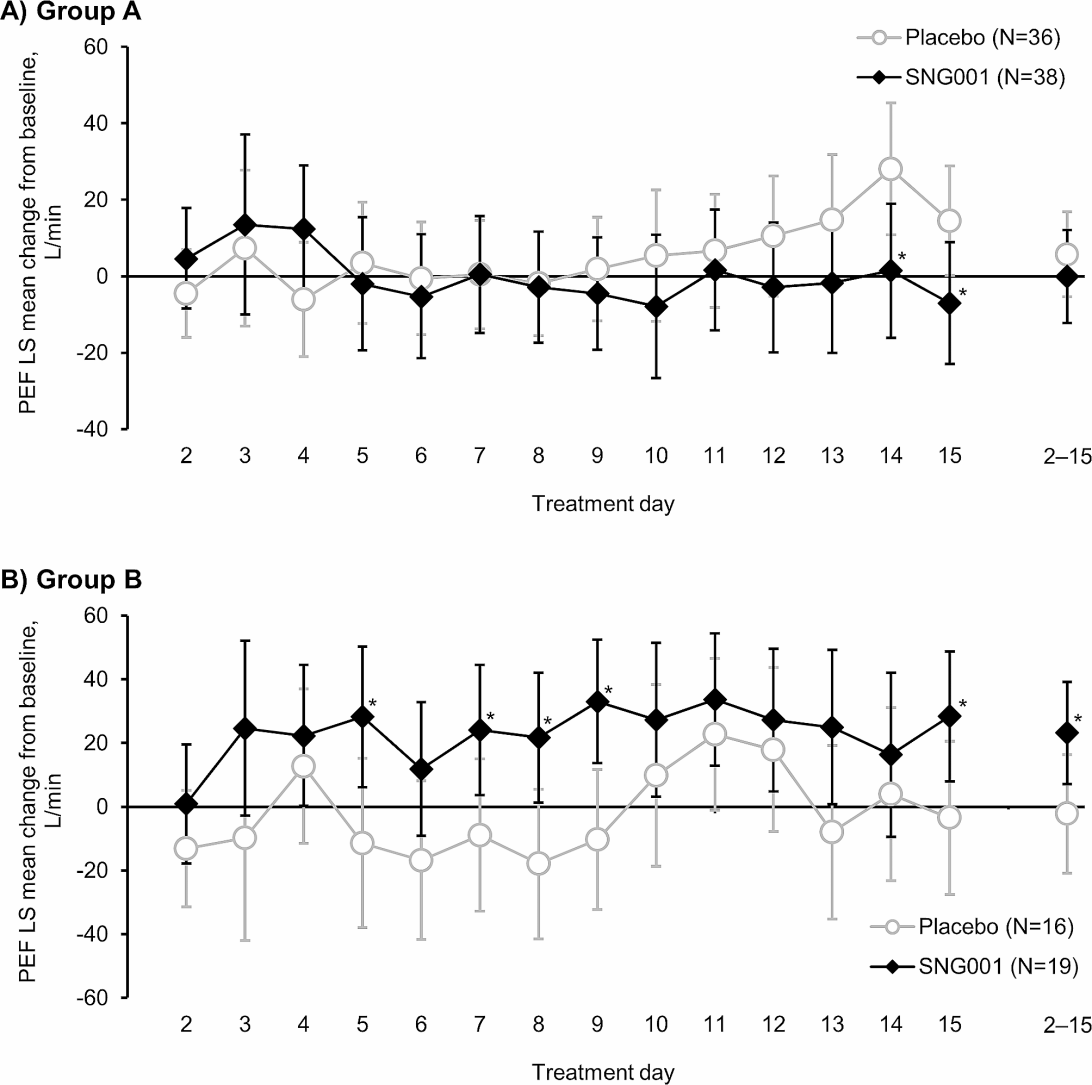
Part 2: Home-assessed PEF change from baseline in (**A**) Group A (cold-like symptoms and/or deterioration in COPD symptoms) and (**B**) Group B (moderate COPD exacerbation) *p < 0.05 SNG001 vs. placebo. Data are least squares mean and 95% confidence interval. Mean (SD) baseline values were 254.8 (96.84), 239.6 (93.21), 212.5 (60.42) and 230.0 (105.71) L/min in placebo Group A, SNG001 Group A, placebo Group B, and SNG001 Group B, respectively. N values are the intention-to-treat population. PEF, peak expiratory flow

For home-assessed PEF in Group A there was no difference between treatments when evaluated over the entire treatment period, although on Days 14 and 15 there were significant differences in favour of placebo (Fig. 4). In contrast, in Group B mean home PEF with SNG001 was significantly better than with placebo, when evaluated over the entire treatment period (SNG001–placebo mean difference 25.5 [95% CI 1.1, 49.9] L/min; *p* = 0.041), and on Days 5, 7–9, and 15. There were no consistent differences between treatments for clinic-assessed FEV_1_, FVC and FEV_1_/FVC, with most changes from baseline being small (Additional file 1: Table S5).

**Fig. 5:**
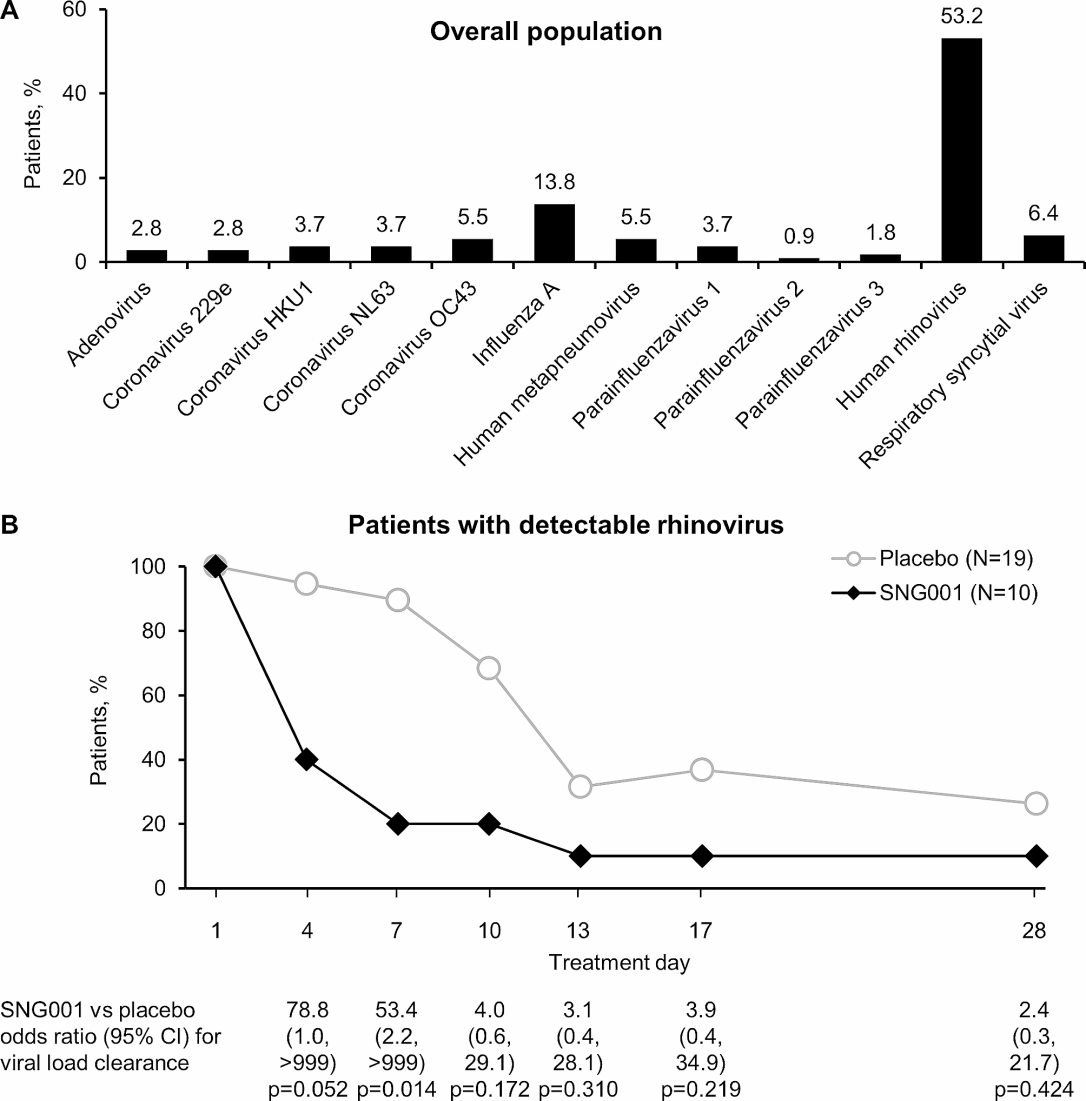
Part 2: (**A**) Summary of baseline positive respiratory virus test results in the overall population; (**B**) Proportion of patients with detectable viral load for rhinovirus at baseline who had detectable rhinovirus viral load at each visit (ITT population) Panel A: Note that patients could test positive for more than one respiratory virus. Panel B: N values are the number of patients with detectable viral load for rhinovirus at baseline

At randomisation (i.e., baseline), patients had a wide range of respiratory viral infections, the most common being human rhinovirus (Fig. 5A and Additional file 1: Figure S3). A *post-hoc* analysis was therefore conducted in the subgroup of patients who had detectable rhinovirus viral load (there were insufficient data for other viruses to perform similar analyses). By Day 4 the proportion of patients receiving SNG001 who had detectable rhinovirus reduced to 40.0% (compared to 94.7% of patients receiving placebo), with a further reduction to 20.0% on Day 7 (versus 89.5% receiving placebo; *p* = 0.014; Fig. 5B).

#### Sputum and serum biomarkers

**Fig. 6:**
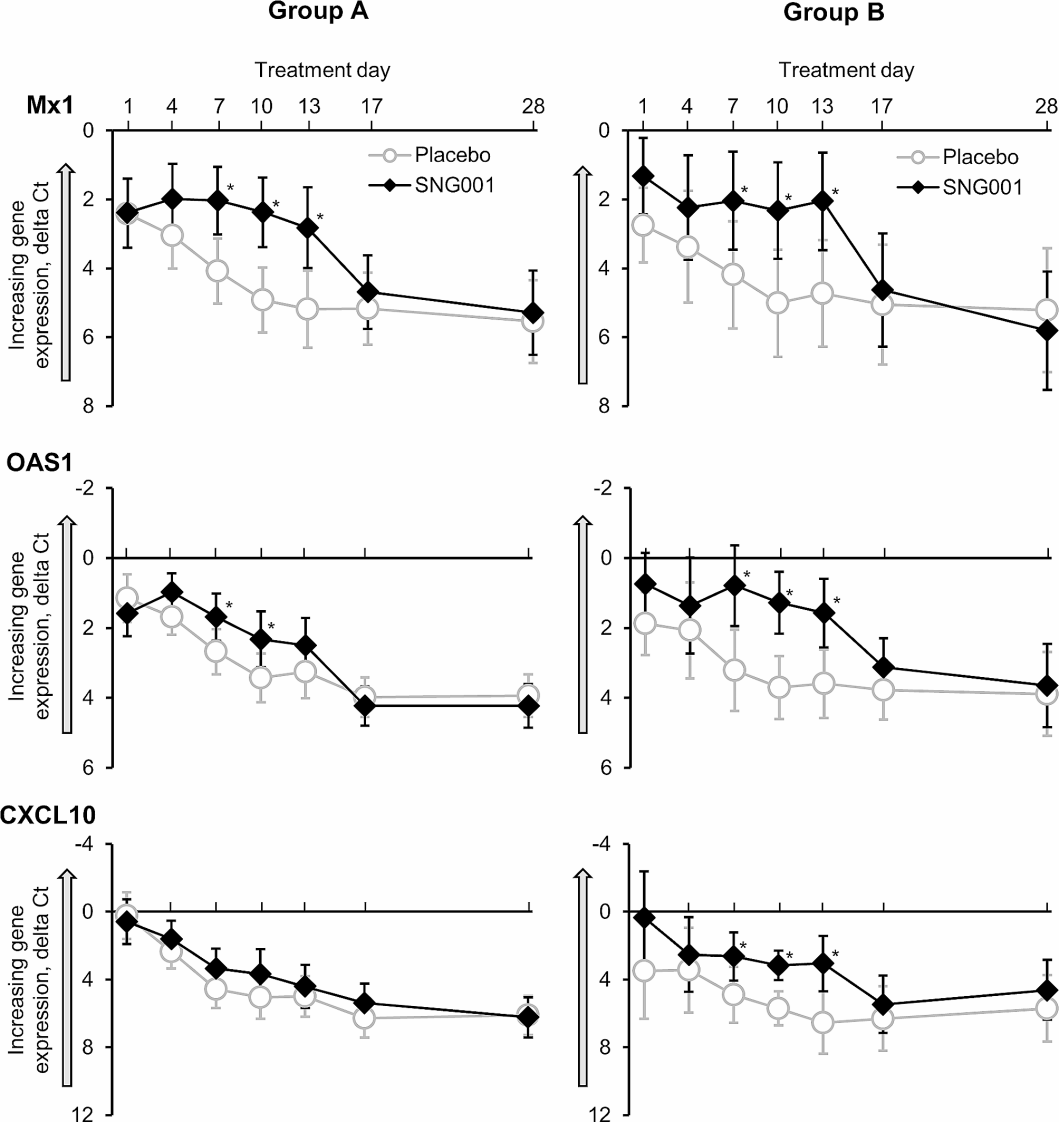
Part 2: Sputum cell interferon-stimulated gene expression in Group A (cold-like symptoms and/or deterioration in COPD symptoms) and Group B (moderate COPD exacerbation) The data plotted are normalised to housekeeping (reference) genes. As reference genes are expressed at a higher level than the interferon-stimulated genes of interest, higher gene expression is indicated by lower values (i.e., in direction of grey arrow). *p < 0.05 SNG001 vs placebo. Data are least squares mean and 95% confidence intervals. Data available at baseline (i.e., pre-dose on Day 1) from 18 and 19 patients receiving placebo and SNG001, respectively, in Group A, and 9 and 7 patients, respectively, in Group B (except for placebo Group B for CXCL10 [N = 8]). Mx1, Mx dynamin like GTPase 1; OAS1, 2’-5’-oligoadenylate synthetase; CXCL10, C-X-C motif chemokine ligand 10

Three of the five sputum ISGs evaluated in Part 1 were evaluated in Part 2 (Mx dynamin like GTPase-1 [Mx1], 2’-5’-oligoadenylate synthetase [OAS1] and CXCL10). For the two ISGs that were not analysed in Part 2, guanylate-binding protein 1 (GBP1) showed lower upregulation than the other ISGs in Part 1, and so was not evaluated in Part 2, and there were issues with the Part 2 assay for interferon-induced protein with tetratricopeptide repeats-2 (IFIT2) that would have necessitated reanalysis, which was not possible due to the COVID-19 pandemic. All three of the evaluated ISGs were upregulated on Day 1 due to the viral infection (Fig. 6). In patients receiving placebo, this initial upregulation subsequently declined; in contrast, expression of these antiviral genes was maintained over the treatment course in patients receiving SNG001, declining by the first assessment in the follow-up period (i.e., Day 17).

Serum levels of interferon-inducible T-cell alpha chemoattractant (ITAC) and CXCL10 increased from baseline (i.e., pre-dose on Day 1) with both treatments in Groups A and B (Additional file 1: Figure S4). Serum levels of the inflammatory gene chemokine (C-C motif) ligand 8 (CCL8) also increased from baseline with both treatments and in both Groups, whereas levels of interleukin-6 (IL-6) were broadly unchanged from baseline (Additional file 1: Figure S5).

**Fig. 7:**
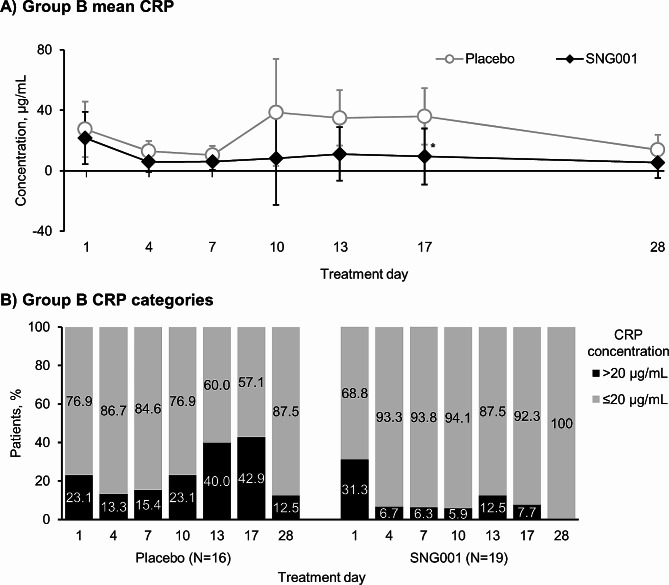
Part 2: (**A**) Mean and (**B**) categorical analyses of serum CRP in Group B (moderate COPD exacerbation) *p < 0.05 SNG001 vs. placebo (there were no statistically significant differences between treatments in the categorical analysis). Panel A: Data are least squares mean and 95% confidence intervals, with data available at baseline (i.e., pre-dose on Day 1) from 13 and 16 patients receiving placebo and SNG001, respectively. CRP, C-reactive protein

**Fig. 8:**
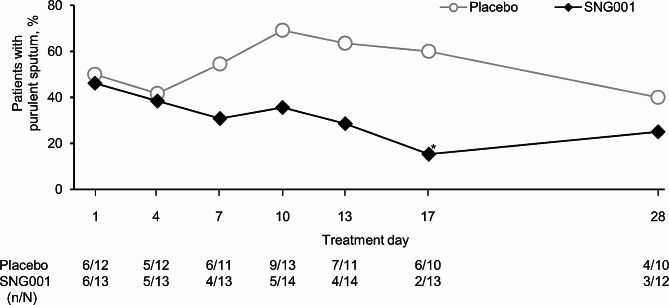
Part 2: Proportion of patients with purulent sputum in Group B (moderate COPD exacerbation) *p < 0.05 SNG001 vs. placebo. Sputum was graded on a colour scale from 1 to 5 where 1 indicated that antibiotics would not be usually required and 5 indicated that antibiotics may be required, with Grades of 3 to 5 reflecting ‘purulent’ sputum. Percentages are calculated as the number of patients with purulent sputum (n) divided by the number with non-missing data (N)

For serum C-reactive protein (CRP), whereas mean changes from baseline in Group A were similar with the two treatments (Additional file 1: Figure S6), for patients in Group B (i.e., those experiencing a moderate COPD exacerbation on entry), levels started to increase from Day 4 with placebo, but were unchanged with SNG001 (Fig. 7A). A *post-hoc* analysis was therefore conducted in Group B, in which CRP levels were categorised by a cut-point of 20 µg/mL at each visit [20]. With placebo, an increasing proportion of patients had CRP > 20 µg/mL from Day 4 to Day 17 (Fig. 7B). In contrast, the proportion of patients receiving SNG001 who had CRP > 20 µg/mL, though higher at baseline (i.e., pre-dose on Day 1), decreased with treatment and remained low throughout follow-up. In Group A, the proportion of patients with purulent sputum was similar with the two treatments (Additional file 1: Figure S7). However, in Group B, whilst the proportion of patients with purulent sputum was similar at baseline, the proportion receiving SNG001 who had purulent sputum decreased over the 14-day treatment period, whereas with placebo the proportion increased from Day 4 to Day 10 before decreasing, resulting in a significant difference between treatments at Day 17 (15.4% with SNG001 vs. 60.0% with placebo; *p* = 0.039; Fig. 8).

#### Safety

Overall, the incidence of adverse events was similar with both treatments and in both Groups (Table 3). None of the events with SNG001 was considered severe, and only one was considered serious (a COPD exacerbation in a patient in Group A, moderate in severity and not considered related to treatment). There were no notable differences in the other safety parameters.

**Table 3:** Part 2: Treatment-emergent adverse events, overall and most common preferred terms (≥ 2 patients with any treatment)

	Group A (cold–like symptoms and/or deterioration in COPD symptoms)		Group B (moderate COPD exacerbation)		Combined	
	Placebo (*N* = 36)	SNG001 (*N* = 38)	Placebo (*N* = 16)	SNG001 (*N* = 19)	Placebo (*N* = 52)	SNG001 (*N* = 57)
Any adverse event	21 (58.3%)	22 (57.9%)	9 (56.3%)	12 (63.2%)	30 (57.7%)	34 (59.6%)
Nasopharyngitis	0	1 (2.6%)	1 (6.3%)	1 (5.3%)	1 (1.9%)	2 (3.5%)
Breast cancer	2 (5.6%)	0	0	0	2 (3.8%)	0
Headache	2 (5.6%)	0	1 (6.3%)	0	3 (5.8%)	0
Flushing	0	1 (2.6%)	0	1 (5.3%)	0	2 (3.5%)
Chronic obstructive pulmonary disease	9 (25.0%)	9 (23.7%)	3 (18.8%)	2 (10.5%)	12 (23.1%)	11 (19.3%)
Wheezing	0	5 (13.2%)	0	0	0	5 (8.8%)
Cough	4 (11.1%)	3 (7.9%)	0	1 (5.3%)	4 (7.7%)	4 (7.0%)
Dyspnoea	3 (8.3%)	2 (5.3%)	0	0	3 (5.8%)	2 (3.5%)
Oropharyngeal pain	2 (5.6%)	0	0	0	2 (3.8%)	0
Diarrhoea	1 (2.8%)	2 (5.3%)	2 (12.5%)	2 (10.5%)	3 (5.8%)	4 (7.0%)
Vessel puncture site bruise	2 (5.6%)	1 (2.6%)	0	0	2 (3.8%)	1 (1.8%)
Any on-treatment adverse event	18 (50.0%)	18 (47.4%)	9 (56.3%)	9 (47.4%)	27 (51.9%)	27 (47.4%)
Nasopharyngitis	0	1 (2.6%)	1 (6.3%)	1 (5.3%)	1 (1.9%)	2 (3.5%)
Flushing	0	1 (2.6%)	0	1 (5.3%)	0	2 (3.5%)
Chronic obstructive pulmonary disease	9 (25.0%)	5 (13.2%)	3 (18.8%)	0	12 (23.1%)	5 (8.8%)
Cough	4 (11.1%)	3 (7.9%)	0	1 (5.3%)	4 (7.7%)	4 (7.0%)
Wheezing	0	3 (7.9%)	0	0	0	3 (5.3%)
Dyspnoea	3 (8.3%)	2 (5.3%)	0	0	3 (5.8%)	2 (3.5%)
Diarrhoea	1 (2.8%)	2 (5.3%)	2 (12.5%)	2 (10.5%)	3 (5.8%)	4 (7.0%)
Vessel puncture site bruise	2 (5.6%)	1 (2.6%)	0	0	2 (3.8%)	1 (1.8%)
Any adverse event related to treatment	7 (19.4%)	5 (13.2%)	6 (37.5%)	4 (21.1%)	13 (25.0%)	9 (15.8%)
Headache	1 (2.8%)	0	1 (6.3%)	0	2 (3.8%)	0
Flushing	0	1 (2.6%)	0	1 (5.3%)	0	2 (3.5%)
Cough	1 (2.8%)	3 (7.9%)	0	1 (5.3%)	1 (1.9%)	4 (7.0%)
Dyspnoea	2 (5.6%)	1 (2.6%)	0	0	2 (3.8%)	1 (1.8%)
Diarrhoea	1 (2.8%)	0	1 (6.3%)	0	2 (3.8%)	0
Any severe adverse event	3 (8.3%)	0	1 (6.3%)	0	4 (7.7%)	0
Breast cancer	2 (5.6%)	0	0	0	2 (3.8%)	0
Any serious adverse event	2 (5.6%)	1 (2.6%)	1 (6.3%)	0	3 (5.8%)	1 (1.8%)
Breast cancer	2 (5.6%)	0	0	0	2 (3.8%)	0
Any serious adverse event related to treatment	0	0	0	0	0	0
Any adverse event leading to discontinuation of study treatment or study withdrawal	1 (2.8%)	0	0	0	1 (1.9%)	0
Any fatal adverse event	0	0	0	0	0	0

## Discussion

Overall, the two parts of this exploratory study demonstrated proof-of-mechanism of SNG001 in patients with COPD, with upregulation of lung antiviral responses in response to inhaled interferon-β that were associated with promotion of viral clearance and a favourable safety and tolerability profile, although with limited effect on the clinical efficacy endpoints (perhaps because use of bronchodilators was permitted throughout the study), aside from an effect on home measured PEF in patients in Group B (who comprised only around a third of the study population). All five assessed ISGs in sputum cells were upregulated following administration of SNG001 in Part 1, with the initial upregulation in Part 2 (in response to the viral infection) prolonged in those patients receiving SNG001, showing that SNG001 boosted lung antiviral responses. Importantly, the fold-changes in sputum Mx1 and OAS1 following administration of SNG001 in Part 1 were consistent with changes in a previous asthma study [21], suggesting that the administered dose is similarly effective in younger patients with asthma and in older patients with COPD – both of whom are at increased risk from viral infections. Furthermore, in Part 2, the *post-hoc* analysis in patients who had a positive test for human rhinovirus (the most commonly detected virus during COPD exacerbations [22]) suggested that viral clearance was enhanced by SNG001 – with a more rapid decrease with SNG001 than placebo. Importantly, SNG001 had a good overall safety and tolerability profile in both parts of the study, with the lack of change in sputum differential cell counts in Part 1 suggesting that SNG001 did not induce an inflammatory response. In particular, there were no reports of fever or ‘flu-like symptoms’ as adverse events. This is consistent with the results of another nebulised formulation of interferon in healthy adults [23], and contrasts with the safety profile of interferon when given by injection [24]. Researchers who conducted a series of in-vitro studies concluded that these symptoms are due to the systemic activity of interferon, which is intrinsically pyrogenic by inducing prostaglandin E2 in the hypothalamus [25]. SNG001 was also similarly well tolerated when administered to patients who had been hospitalised with COVID-19 in previous studies [14, 15]. Overall, the results of these various studies support the potential for nebulised delivery of interferon.

The most intriguing data in Part 2 were for patients in Group B (i.e., those with a moderate COPD exacerbation). In this Group, patients receiving placebo had an increase in CRP in the second week of the treatment period (Days 10–17) that, when combined with the increased incidence of purulent sputum, potentially suggests the presence of a secondary bacterial infection [26, 27]. This was not the case in patients receiving SNG001. Taken together, these data suggest that SNG001 accelerates viral clearance in the lung, and that in patients with a moderate COPD exacerbation it may have the potential to prevent secondary bacterial infections [6]. Given the study was conducted between February 2018 and May 2020, few of the patients would have been active in the study during the COVID-19 pandemic, and although a number of patients tested positive for one of the coronaviruses, none were severe acute respiratory syndrome coronavirus 2 (SARS-CoV-2). We therefore cannot draw any conclusions about the link between SARS-CoV-2 and COPD exacerbations.

The main limitation of the results is that although detailed data are available on viral load in Part 2, data are not available on bacterial load – and therefore the hypothesis that SNG001 may prevent secondary bacterial infections is supported only by indirect data, specifically serum CRP and sputum colour. In addition, both parts of the study recruited relatively small numbers of patients. In particular, the patients of most interest in Part 2 were those who had a moderate COPD exacerbation. However, this comprised only 32% of the overall population, and it would be interesting to evaluate the efficacy of SNG001 specifically in this patient population in an appropriately powered and designed study. Moreover, although sputum is a useful way of assessing treatment efficacy directly within the lung, sputum samples were not available for all patients at all visits. Furthermore, we could only study clearance of human rhinovirus, as the other viruses were insufficiently prevalent – to evaluate the effect of SNG001 on other viruses would require a much larger study. Additionally, from the data available in Part 2 we cannot elucidate the impact of smoking status on the disease course from any impact on treatment efficacy. Finally, although considered sufficient for the purposes of the study, a small number of patients were included in Part 1, the majority of whom (77%) received SNG001.

## Conclusions

This exploratory study provides evidence that the recombinant interferon-β1a SNG001 is well tolerated in patients with COPD, and upregulates the lung’s antiviral defences to accelerate viral clearance. In particular in patients with a moderate COPD exacerbation, there was evidence that SNG001 provided an improvement in PEF and potentially prevented secondary bacterial infection. These findings warrant investigation in a larger study – especially one that includes an evaluation of bacterial load, either by culture or molecular methods.

### Electronic supplementary material

Below is the link to the electronic supplementary material.

Supplementary Material 1

## Data Availability

The data analysed and presented in this study are available from the corresponding author on reasonable request, providing the request meets local ethical and research governance criteria after publication. Patient-level data will be anonymised and study documents will be redacted to protect the privacy of trial participants.

## Abbreviations

BCSS: Breathlessness, Cough and Sputum Scale
BMI: body-mass index
CAT: COPD Assessment Test
CCL8: chemokine (C-C motif) ligand 8
COVID-19: Coronavirus Disease 2019
CRP: C-reactive protein
CXCL10: C-X-C motif chemokine ligand-10
FEV_1_: forced expiratory volume in 1 s
GBP1: guanylate-binding protein 1
IFIT2: interferon-induced protein with tetratricopeptide repeats-2
IL-6: interleukin-6
ISG: interferon-stimulated gene
ITAC: interferon-inducible T-cell alpha chemoattractant
MMRM: mixed model for repeated measures
Mx1: MX dynamin-like GTPase-1
NC: not calculated
OAS1: 2’-5’-oligoadenylate synthetase
PEF: peak expiratory flow
SARS-CoV-2: severe acute respiratory syndrome coronavirus 2

## References

[1] Global Initiative for Chronic Obstructive Lung Disease. Global strategy for the diagnosis, management, and prevention of chronic obstructive pulmonary disease [Internet]. 2024 [cited 2024 Mar 18]. https://goldcopd.org/2024-gold-report/

[2] Seemungal TA, Donaldson GC, Bhowmik A, Jeffries DJ, Wedzicha JA (2000). Time course and recovery of exacerbations in patients with chronic obstructive pulmonary disease. Am J Respir Crit Care Med.

[3] Mallia P, Johnston SL (2006). How viral infections cause exacerbation of airway diseases. Chest.

[4] Linden D, Guo-Parke H, Coyle PV, Fairley D, McAuley DF, Taggart CC (2019). Respiratory viral infection: a potential missing link in the pathogenesis of COPD. Eur Respir Rev.

[5] Tan K, Sen, Lim RL, Liu J, Ong HH, Tan VJ, Lim HF (2020). Respiratory viral infections in exacerbation of chronic airway inflammatory diseases: novel mechanisms and insights from the upper airway epithelium. Front Cell Dev Biol.

[6] Mallia P, Footitt J, Sotero R, Jepson A, Contoli M, Trujillo-Torralbo MB (2012). Rhinovirus infection induces degradation of antimicrobial peptides and secondary bacterial infection in chronic obstructive pulmonary disease. Am J Respir Crit Care Med.

[7] Yang E, Li MMH (2020). All about the RNA: Interferon-stimulated genes that interfere with viral RNA processes. Front Immunol.

[8] Fox LE, Locke MC, Lenschow DJ (2020). Context is key: delineating the unique functions of IFNα and IFNβ in disease. Front Immunol.

[9] Kali SK, Dröge P, Murugan P (2021). Interferon β, an enhancer of the innate immune response against SARS-CoV-2 infection. Microb Pathog.

[10] Hilzendeger C, da Silva J, Henket M, Schleich F, Corhay JL, Kebadze T (2016). Reduced sputum expression of interferon-stimulated genes in severe COPD. Int J Chron Obstruct Pulmon Dis.

[11] Mallia P, Message SD, Gielen V, Contoli M, Gray K, Kebadze T (2011). Experimental rhinovirus infection as a human model of chronic obstructive pulmonary disease exacerbation. Am J Respir Crit Care Med.

[12] Djukanović R, Harrison T, Johnston SL, Gabbay F, Wark P, Thomson NC (2014). The effect of inhaled IFN-β on worsening of asthma symptoms caused by viral infections. A randomized trial. Am J Respir Crit Care Med.

[13] McCrae C, Olsson M, Gustafson P, Malmgren A, Aurell M, Fagerås M (2021). INEXAS: a phase 2 randomized trial of on-demand inhaled interferon beta-1a in severe asthmatics. Clin Exp Allergy.

[14] Monk PD, Marsden RJ, Tear VJ, Brookes J, Batten TN, Mankowski M (2021). Safety and efficacy of inhaled nebulised interferon beta-1a (SNG001) for treatment of SARS-CoV-2 infection: a randomised, double-blind, placebo-controlled, phase 2 trial. Lancet Respir Med.

[15] Monk PD, Brookes JL, Tear VJ, Batten TN, Mankowski M, Adzic-Vukicevic T (2023). Nebulised interferon beta-1a (SNG001) in hospitalised COVID-19: SPRINTER phase III study. ERJ Open Res.

[16] Ye J, Chen J (2021). Interferon and Hepatitis B: current and future perspectives. Front Immunol.

[17] Reynolds S, Lunn K, Beegan R, Tear V, Monk PD (2019). Antiviral biomarkers are upregulated in sputum cells following administration of inhaled interferon beta to COPD patients. Eur Respir J.

[18] Leidy NK, Rennard SI, Schmier J, Jones MKC, Goldman M (2003). The breathlessness, Cough, and Sputum Scale. The development of empirically based guidelines for interpretation. Chest.

[19] Leidy NK, Schmier JK, Jones MKC, Lloyd J, Rocchiccioli K (2003). Evaluating symptoms in chronic obstructive pulmonary disease: validation of the breathlessness, cough and sputum scale. Respir Med.

[20] Escadafal C, Incardona S, Fernandez-Carballo BL, Dittrich S (2020). The good and the bad: using C reactive protein to distinguish bacterial from non-bacterial infection among febrile patients in low-resource settings. BMJ Glob Heal.

[21] Monk PD, Reynolds S, Lunn K, Beegan R, Roberts J, Tear VJ (2019). Upregulation of antiviral biomarkers in sputum cells following administration of inhaled interferon beta to patients with COPD. Am J Respir Crit Care Med.

[22] Zwaans WAR, Mallia P, van Winden MEC, Rohde GGU (2014). The relevance of respiratory viral infections in the exacerbations of chronic obstructive pulmonary disease—a systematic review. J Clin Virol.

[23] Garcia-Huidobro D, Iturriaga C, Perez-Mateluna G, Fajuri P, Severino N, Urzúa M (2023). Safety, tolerability, bioavailability, and biological activity of inhaled interferon-α2b in healthy adults: the IN2COVID Phase I randomized trial. Clin Drug Investig.

[24] Reder AT, Oger JF, Kappos L, O’Connor P, Rametta M (2014). Short-term and long-term safety and tolerability of interferon β-1b in multiple sclerosis. Mult Scler Relat Disord.

[25] Dinarello CA, Bernheim HA, Duff GW, Le HV, Nagabhushan TL, Hamilton NC (1984). Mechanisms of fever induced by recombinant human interferon. J Clin Invest.

[26] Francis NA, Gillespie D, Wootton M, White P, Bates J, Richards J (2020). Clinical features and C-reactive protein as predictors of bacterial exacerbations of COPD. Int J Chron Obstruct Pulmon Dis.

[27] Hoult G, Gillespie D, Wilkinson TMA, Thomas M, Francis NA (2022). Biomarkers to guide the use of antibiotics for acute exacerbations of COPD (AECOPD): a systematic review and meta-analysis. BMC Pulm Med.

