# Supplementary material for: Nebulised interferon beta-1a (SNG001) in the treatment of viral exacerbations of COPD
Source: Respir Res. 2024 May 29;25:228. doi: 10.1186/s12931-024-02854-7 (PMC11138078; doi:10.1186/s12931-024-02854-7)
Supplement: Supplementary file 1 — Supplementary Material 1 [file 12931_2024_2854_MOESM1_ESM.docx]

# Nebulised interferon beta-1a (SNG001) in the treatment of viral exacerbations of COPD

Phillip D Monk PhD, Jody L Brookes BSc, Victoria J Tear PhD, Toby N Batten MSc, Clare Newall PhD, Marcin Mankowski MD, Michael G Crooks MD, Dave Singh MD, Rekha Chaudhuri MD, Brian Leaker DM, Kerry Lunn, Sophie Reynolds PhD, Sarah Dudley PhD, Felicity J Gabbay FMedSci, Stephen T Holgate FMedSci, Ratko Djukanovic MD, Thomas MA Wilkinson PhD, on behalf of the SG015 study group

# Additional File 1

# Methods

## Part 1

### Sputum samples

Sputum was induced according to the site’s standard operating procedure, and was processed within 2 h of collection. At the screening visit, a minimum of 0.1 g of sputum was required for the patient to be eligible, with squamous cell contamination ≤30% and viable leukocyte count ≥40%. At other visits, if <0.05 g was collected, the sample was discarded. Between 0.1 and 0.4g of the sputum was mixed with 0.1% Sputolysin and filtered, with supernatant and cells separated by centrifugation. The cells were counted and used to make two cytospins, with the remainder lysed in RNA Lysis Buffer (RLT) and stored. The cytospins were fixed, stained with a differential cell stain (Tryptan Blue), and coverslipped, and differential cell counts were carried out using a haemocytometer viewed using a microscope.

If >0.4 g of sputum was collected, the remainder was stored as a plug; this was placed into a 2 mL tube, RLT buffer was added, and the tube was vortexed to mix the plug and buffer. To assess antiviral biomarkers, messenger ribonucleic acid (mRNA) was isolated from either lysed sputum cells or sputum plugs using commercially available spin column kits. Copy deoxyribose nucleic acid (cDNA) was produced by a reverse transcription reaction, with antiviral genes and reference genes then measured by reverse transcriptase polymerase chain reaction.

### Inclusion Criteria

Patients must have met all of the following inclusion criteria at screening and pre-dose to be eligible for randomisation:

1. Male or female, 40–75 years of age at the time of the screening visit.
2. A confirmed physician diagnosis of COPD or a medical history consistent with a diagnosis of COPD for at least 12 months prior to the screening visit.
3. Post-bronchodilator FEV_1_ ≥40% predicted and FEV_1_/FVC ratio <0.7 (at screening).
4. FEV_1_ ≥30% predicted (at Visit 2, pre-dose).
5. Stable COPD, having no symptoms of an exacerbation and/or respiratory tract infection currently and/or within the past 6 weeks of screening and/or randomisation.
6. Prescribed and taking regularly one or more long acting bronchodilators (e.g. long acting β_2_ agonist [LABA], long acting muscarinic antagonist [LAMA]) with or without an inhaled corticosteroid maintenance therapy for their COPD.
7. Produce sputum most days.
8. Provide written informed consent.
9. Produce an adequate sputum sample at the screening visit.
10. Female patients must have been 1 year post-menopausal, surgically sterile, or using an acceptable method of contraception. Acceptable birth control methods were tubal occlusion, intrauterine device (provided coils are copper-banded), levonorgestrel intrauterine system, medroxyprogesterone injections, etonogestrel implants, normal and low dose combined oral pills, norelgestromin / ethinylestradiol transdermal system, intravaginal device, desogestrel, total sexual abstinence and vasectomised sexual partner. Women should have been stable on their chosen method of birth control for a minimum of three months before entering the trial and should continue with birth control for one month after the last dose of inhaled interferon-β-1a/matching placebo. In addition to the acceptable birth control method (except for the practice of total sexual abstinence), condom (with spermicides) should have been used by the male partner for sexual intercourse from randomisation (Visit 2) and for 1 month after the last dose of inhaled interferon-β-1a/matching placebo to prevent pregnancy. Women of childbearing potential must have a negative pregnancy test at screening and prior to randomisation. Women not of childbearing potential are defined as women who are either permanently sterilised (hysterectomy, bilateral oophorectomy, or bilateral salpingectomy), or who are postmenopausal. Women will be considered postmenopausal if they have been amenorrhoeic for 12 months prior to the planned date of randomisation without an alternative medical cause. The following age specific requirements applied: Women <50 years old would be considered postmenopausal if they have been amenorrhoeic for 12 months or more following cessation of exogenous hormonal treatment and if follicle stimulating hormone (FSH) levels are in the postmenopausal range. If the FSH result was not available at the time of randomisation, the patient must have a negative pregnancy test and agree to use highly effective contraception methods until the FSH result was available; Women ≥50 years old were considered postmenopausal if they had been amenorrhoeic for 12 months or more following cessation of all exogenous hormonal treatment.
11. Motivation (in the investigator’s opinion) to comply with protocol requirements and complete all study visits, including the ability to communicate well with the Investigator and be capable of understanding the nature of the research and its treatment (including its risks and potential benefits).

### Exclusion Criteria

A patient must not have been randomised into the study if they met any of the following criteria at screening and pre-dose:

1. Any condition, including findings in the medical history or in the pre-randomisation assessments that in the opinion of the Investigator, constituted a risk or a contraindication for the participation of the patient in the study or that could interfere with the study objectives, conduct or evaluation.
2. Current treatment or treatment within the past 6 weeks with oral corticosteroids.
3. Oxygen saturation ≤92%.
4. Required any form of oxygen therapy or non-invasive ventilation.
5. Had received live/attenuated vaccines in the six weeks prior to randomisation or inactivated/killed, subunit or conjugate vaccines in the two weeks prior to randomisation.
6. Current or previous participation in another clinical trial where the patient received a dose of an investigational medicinal product (IMP) containing small molecules within 12 weeks prior to entry into this study or containing biologicals within 12 months prior to entry.
7. Active interstitial lung disease or past history of lung cancer not considered cured, significant bronchiectasis, cystic fibrosis, alpha-1 antitrypsin deficiency or a history of significant chronic asthma.
8. Currently have, or had within the past 3 months, any significant underlying medical condition(s) that could impact the interpretation of results (e.g. non respiratory infections, haematologic disease, malignancy, renal disease, hepatic disease, coronary heart disease or other cardiovascular disease [including arrhythmias], endocrine or gastrointestinal disease).
9. History of hypersensitivity to natural or recombinant interferon-β or to any of the excipients in the drug preparation.
10. Significant history of depressive disorder or suicidal ideation. Specifically, individuals with current severe depression (i.e. a low mood, which pervades all aspects of life and an inability to experience pleasure in activities that formerly were enjoyed); individuals with a past history of depression that required hospitalisation or referral to psychiatric services in the past 5 years; individuals who currently feel suicidal or have attempted suicide in the past.
11. Currently receiving anti-epileptic therapy and/or have uncontrolled epilepsy.
12. History of drug or alcohol abuse within 12 months prior to enrolment.
13. Female who is breast-feeding, pregnant or intends to become pregnant.
14. Clinically significant arrhythmias or implantation of permanent pacemaker or implanted cardiac defibrillator.
15. Unstable ischemic heart disease (including, but not limited to, unstable angina or myocardial infarction) or stroke within the preceding six months.

## Part 2

### Sputum viral load and biomarkers

To confirm the presence of a respiratory virus infection (so that patients could be included in the study), and to determine sputum viral load, sputum or a swab was tested with the BioFire Respiratory panel (bioMerieux, Marcy-l'Étoile, France), which is a multiplex rapid polymerase chain reaction (PCR) test. A positive result was determined by one or more virus(es) being detected on the machine:

- Coronavirus HKU1
- Coronavirus NL63
- Coronavirus 229E
- Coronavirus OC43
- Human rhinovirus/enterovirus
- Influenza A
- Influenza A/H1
- Influenza A/H3
- Influenza A/H1-2009
- Influenza B
- Parainfluenza virus 1
- Parainfluenza virus 2
- Parainfluenza virus 3
- Parainfluenza virus 4
- Respiratory syncytial virus

For sputum collection, sites were chosen based on their experience in selecting sputum plugs. Total RNA was extracted from 0.1–0.2g sputum plugs by lysing in 1.2 mL RLT buffer and homogenising through QIAshredder columns. Samples were extracted using an RNeasy mini kit. The resulting RNA was DNase treated with the Invitrogen Turbo DNA-free kit to remove any genomic DNA, and reverse transcribed with a PrimerDesign Precision nanoScript 2 reverse transcription (RT) kit. Viral load was quantified using cDNA from the RT with Genesig real-time PCR detection kits.

To quantify sputum biomarkers, PCR was performed on cDNA for antiviral biomarkers in duplicate for each patient sample. Reference gene GAPDH and genes of interest CXCL10, MX1, OAS1 were measured.

### Serum biomarkers

Approximately 10 mL of blood was collected at each visit. Tubes were left to clot for 30–45 minutes before centrifuging at 4 C for 10 min at 2500xg. The serum layer was removed from the tube into a 15 mL tube and inverted several times to mix. Serum biomarker measurement was carried out using the Simple Protein Ella Automated Immunoassay System, with individual cartridges used for each analyte.

### Pre-treatment inclusion criteria

Patients must have met all of the following inclusion criteria at the consent/screening visit (Visit 1) to be eligible for enrolment into the study:

1. Male or female, 40–85 years of age at the time of the consent/screening visit.
2. A confirmed physician diagnosis of COPD or a medical history consistent with a diagnosis of COPD for at least 12 months prior to the Consent/Screening Visit.
3. Current or ex-smoker with ≥10 pack years of smoking history.
4. Post-bronchodilator FEV_1_/FVC ratio <0.7.
5. Post-bronchodilator FEV_1_ ≥30% of the predicted value.*
6. History of ≥1 COPD exacerbations in the last 12 months requiring intervention with oral corticosteroids and/or antibiotics.
7. Patient-reported evidence that a respiratory virus has made their COPD significantly worse in the past.
8. Prescribed and taking regularly one or more long acting bronchodilator (e.g. LABA, LAMA) with or without an inhaled corticosteroid maintenance therapy for their COPD.
9. Patients on self-management plans agree to consult a healthcare professional prior to taking oral corticosteroids or antibiotics for treatment of a COPD exacerbation.
10. Provide written informed consent.
11. Be the owner of a mobile phone, and be able to, and agree to, respond to the required SMS (text) messages for the trial.
12. Female patients must have been one-year post-menopausal, surgically sterile, or using an acceptable method of contraception. Acceptable birth control methods were tubal occlusion, intrauterine device (provided coils are copper-banded), levonorgestrel intrauterine system, medroxyprogesterone injections, etonogestrel implants, normal and low dose combined oral pills, norelgestromin / ethinylestradiol transdermal system, intravaginal device, desogestrel, total sexual abstinence and vasectomised sexual partner. Women should have been stable on their chosen method of birth control for a minimum of three months before entering the trial and should continue with birth control for one month after the last dose of inhaled interferon-β-1a/matching placebo. In addition to the acceptable birth control method (except for the practice of total sexual abstinence), condom (in UK with spermicides) should be used by the male partner for sexual intercourse from randomisation (Visit 2) and for 1 month after the last dose of inhaled interferon-β-1a/matching placebo to prevent pregnancy. Women not of childbearing potential were defined as women either permanently sterilised (hysterectomy, bilateral oophorectomy, or bilateral salpingectomy), or who are postmenopausal. Women were considered postmenopausal if they had been amenorrhoeic for 12 months prior to the planned date of randomisation without an alternative medical cause. The following age specific requirements applied: Women <50 years old would be considered postmenopausal if they have been amenorrhoeic for 12 months or more following cessation of exogenous hormonal treatment and if FSH levels are in the postmenopausal range; Women ≥50 years old would be considered postmenopausal if they have been amenorrhoeic for 12 months or more following cessation of all exogenous hormonal treatment.
13. Motivation (in the Investigator’s opinion) to comply with protocol requirements and complete all study visits, including the ability to communicate well with the Investigator and be capable of understanding the nature of the research and its treatment (including its risks and potential benefits).

*The first 16 patients included in the study had FEV_1_ ≥40% of the predicted value. The safety data were reviewed by the DSMC and the criterion was changed to post-bronchodilator FEV_1_ ≥30% of the predicted value.

### Pre-treatment exclusion criteria

A patient must not have been enrolled into the study if they met any of the following criteria:

1. Any condition, including findings in the medical history or in the pre-study assessments, or any treatment, that in the opinion of the Investigator, constituted a risk or a contraindication for the participation of the patient in the study or that could interfere with the study objectives, conduct or evaluation.
2. Current moderate or severe exacerbation of COPD.
3. Moderate or severe exacerbation of COPD that resolved less than two weeks prior (with resolution defined as return to patient’s baseline COPD symptoms or the Investigator does not expect any further improvement of patient’s symptoms).
4. Stopped taking treatment (antibiotics and/or oral corticosteroids) for an exacerbation of COPD less than two weeks prior.
5. Currently had an upper or lower respiratory tract infection.
6. Oxygen saturation ≤92%.
7. Required long-term oxygen therapy.
8. Current or previous participation in another clinical trial where the patient received small molecules within 30 days or five half-lives (whichever was longer) prior to entry into this study or containing biologicals within three months prior to entry.
9. Active interstitial lung disease or past history of lung cancer not considered cured, significant bronchiectasis, cystic fibrosis, alpha-1 antitrypsin deficiency or a history of significant chronic asthma.
10. Currently have, or had within the past 3 months, any significant underlying medical condition(s) that could impact interpretation of results (e.g. non respiratory infections, haematologic disease, malignancy, renal disease, hepatic disease, coronary heart disease or other cardiovascular disease [including arrhythmias], endocrine or gastrointestinal disease).
11. History of hypersensitivity to natural or recombinant interferon-β or to any of the excipients in the drug preparation.
12. Significant history of depressive disorder or suicidal ideation. Specifically, individuals with current severe depression (i.e. a low mood, which pervades all aspects of life and an inability to experience pleasure in activities that formerly were enjoyed); individuals with a past history of depression that required hospitalisation or referral to psychiatric services in the past 5 years; individuals who currently feel suicidal or have attempted suicide in the past 5 years.
13. Currently receiving anti-epileptic therapy and/or have uncontrolled epilepsy.
14. History of drug or alcohol abuse within 12 months prior to enrolment .
15. Female who is breast-feeding, lactating, pregnant or intends to become pregnant.
16. Clinically significant arrhythmias or implantation of permanent pacemaker or implanted cardiac defibrillator.
17. Unstable ischaemic heart disease (including, but not limited to, unstable angina or myocardial infarction) or stroke within the preceding six months.

### Treatment Inclusion Criteria

To be eligible for randomisation into the treatment phase of the study, patients must have met the following criteria:

1. One of the following:
   - Symptoms of an upper tract respiratory infection which started within the 48 h prior to first dose (Group A); OR
   - Deterioration in COPD symptoms with or without symptoms of an upper respiratory tract infection which started within the 48 h prior to first dose (Group A); OR
   - Moderate exacerbation of COPD with or without symptoms of an upper respiratory tract infection which started within the 48 h prior to first dose (Group B). A moderate exacerbation was defined according to the Global Initiative for Chronic Obstructive Lung Disease 2017 guidelines as an acute worsening of respiratory symptoms that results in additional therapy treated with short-acting bronchodilators plus antibiotics and/or oral corticosteroids.
2. Positive for respiratory virus.
3. Continued to use their regular COPD medication since the Consent/Screening Visit.
4. Post-bronchodilator FEV_1_ ≥30% of the predicted value.*
5. Motivated (in the investigator’s opinion) and available to complete all study visits, and had the ability to communicate well with the investigator and be capable of understanding the nature of the research and its treatment (including its risks and benefits).
6. Females of childbearing potential must have been using an effective form of birth control and agree to maintain this usage throughout the duration of the treatment phase of the study and for four weeks after the last dose of study drug. Females of childbearing potential must have had a negative pregnancy test prior to randomisation. Women <50 years old who considered postmenopausal due to being amenorrhoeic for ≥12 months following cessation of exogenous hormonal treatment had to have a confirmation of follicle stimulating hormone (FSH) levels in the postmenopausal range. If the FSH result was not available at the time of randomisation, the patient must have had a negative pregnancy test and agree to use highly effective contraception methods until the FSH result was available.

*The first 16 patients included in the study had FEV_1_ ≥40% of the predicted value. The safety data were reviewed by the DSMC and the criterion was changed to post-bronchodilator FEV_1_ ≥30% of the predicted value.

### Treatment Exclusion Criteria

Patients may not have been randomised into the treatment phase if they met any of the following exclusion criteria:

1. Currently has a severe exacerbation of COPD.
2. Had a moderate or severe COPD exacerbation that resolved less than two weeks prior (with resolution defined as return to patient’s baseline COPD symptoms or the Investigator does not expect any further improvement of patient’s symptoms).
3. Stopped taking treatment (antibiotics and/or oral corticosteroids) for the previous exacerbation of COPD less than two weeks prior.
4. Upper or lower respiratory tract infection that resolved less than two weeks prior.
5. The patient had received live/attenuated vaccines in the six weeks prior to randomisation or inactivated/killed, subunit or conjugate vaccines in the two weeks prior to randomisation.
6. Diagnosed with any further lung disease since the Consent/Screening Visit.
7. Took oral corticosteroids and/or antibiotics for >48 hours for a COPD exacerbation prior to dosing.
8. Initiated treatment with roflumilast or a macrolide in the four weeks prior to randomisation.
9. Positive virus test, but in the Investigator’s opinion did not present with any cold symptoms or did not present with a worsening of COPD symptoms assessed as a moderate exacerbation.
10. Any condition, including findings in the medical history, or was receiving any treatment that in the opinion of the investigator, constituted a risk or a contraindication for the participation of the patient into the study or that could interfere with the study objectives, conduct or evaluation during the treatment phase.
11. Since the consent/screening visit, taken or started to take any medication other than their COPD medications that the investigator deemed not suitable for inclusion into the treatment phase.
12. Breast feeding, lactating or pregnant.
13. Current or previous participation in another clinical trial where the patient received a dose of an IMP containing small molecules within 30 days or five half-lives (whichever is longer) prior to entry into this study or containing biologicals within three months prior to entry into this study.

## Individual patient stopping rules

In addition to standard clinical study stopping rules (e.g., withdrawal of consent, experiencing intolerable adverse events), the following individual stopping rules were pre-specified for Part 1 of the study:

- The patient’s clinic FEV1 post-dose value decreased by 20% or more below the pre-dose value measured at the baseline visit or Day 2 and, in the Investigator’s opinion, this was associated with clinically significant signs or symptoms, such as breathlessness.
- The patient’s clinic FEV1 pre-dose value at Day 2 or Day 3 was below 30% of predicted.
- The patient acquired an upper respiratory/lower respiratory tract infection at any time.

# Results – Part 1

Table S1. Part 1: Statistical analysis of antiviral interferon-stimulated gene expression on Days 2 and 4 following administration of SNG001 compared to baseline.

| **Gene** | **Baseline, mean** | **Least squares mean change from baseline (95% CI)** | |
| --- | --- | --- | --- |
|  |  | **Day 2** | **Day 4** |
| MX dynamin-like GTPase-1 (Mx1) | 3.96 | –3.01 (–3.65, –2.37); p<0.0001 | –3.50 (–4.18, –2.83); p<0.0001 |
| 2'-5'-oligoadenylate synthetase (OAS1) | 2.68 | –2.37 (–2.79, –1.94); p<0.0001 | –2.44 (–2.94, –1.94); p<0.0001 |
| C-X-C motif chemokine ligand-10 (CXCL-10) | 1.77 | –3.17 (–5.51, –0.83); p=0.0195 | –2.20 (–4.53, 0.14); p=0.0596 |
| Interferon-induced protein with tetratricopeptide repeats-2 (IFIT2) | –0.20 | –3.48 (–4.44, –2.53); p=0.0001 | –3.62 (–4.74, –2.49); p=0.0003 |
| Guanylate-binding protein 1 (GBP1) | –0.24 | –1.19 (–1.69, –0.70); p=0.0011 | –1.19 (–1.72, –0.67); p=0.0012 |

Data available from 6 and 5 patients on Days 2 and 4, respectively, for Mx1, CXCL10, and GBP1, and for 5 and 4 patients, respectively, for OAS1 and IFIT2. Values were not calculated for placebo, given data were available for ≤3 patients.

Table S2. Part 1: Sputum differential cell count following administration of SNG001.

| **Cell type, 10^6^ cells/g** | **Baseline, mean (SD)** | **Mean change from baseline (SD)** | |
| --- | --- | --- | --- |
|  |  | **Day 2** | **Day 4** |
| Neutrophils | 3.233 (2.9230) | 0.713 (1.9461) | 0.155 (1.2344) |
| Macrophages | 0.961 (1.0394) | 0.405 (0.8437) | –0.098 (0.3563) |
| Eosinophils | 0.185 (0.2055) | 0.120 (0.1925) | 0.056 (0.1320) |
| Lymphocytes | 0.007 (0.0117) | –0.005 (0.0074) | –0.004 (0.0062) |
| Epithelial | 0.058 (0.0275) | 0.047 (0.0469) | 0.029 (0.0862) |

Data available from 7 and 6 patients on Days 2 and 4, respectively.

Figure S1. Part 1: FEV_1_ change from baseline during the treatment period.


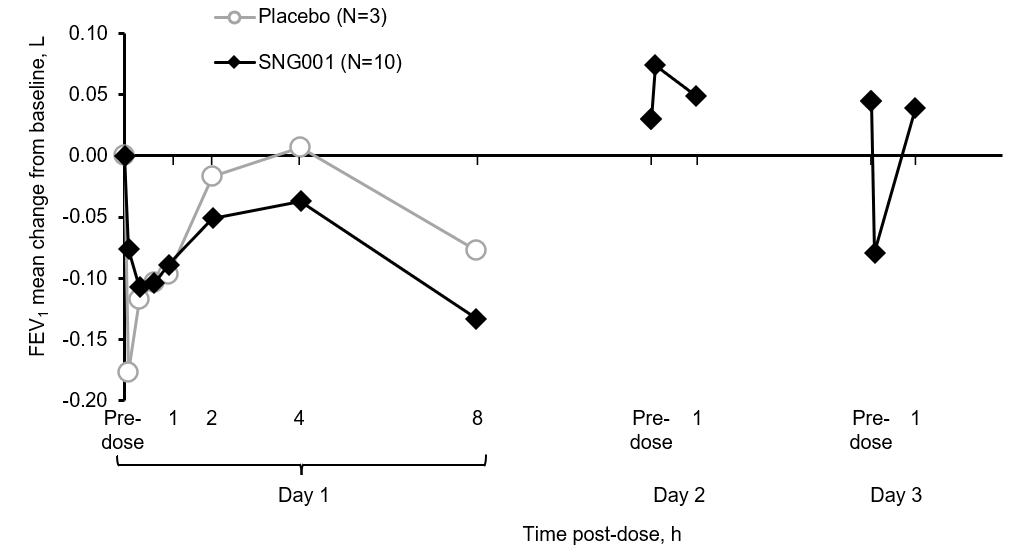


FEV_1_, forced expiratory volume in 1 sec. Mean changes from baseline were not calculated for placebo on Days 2 or 3.

Figure S2. Part 1: PEF change from baseline during the treatment period.


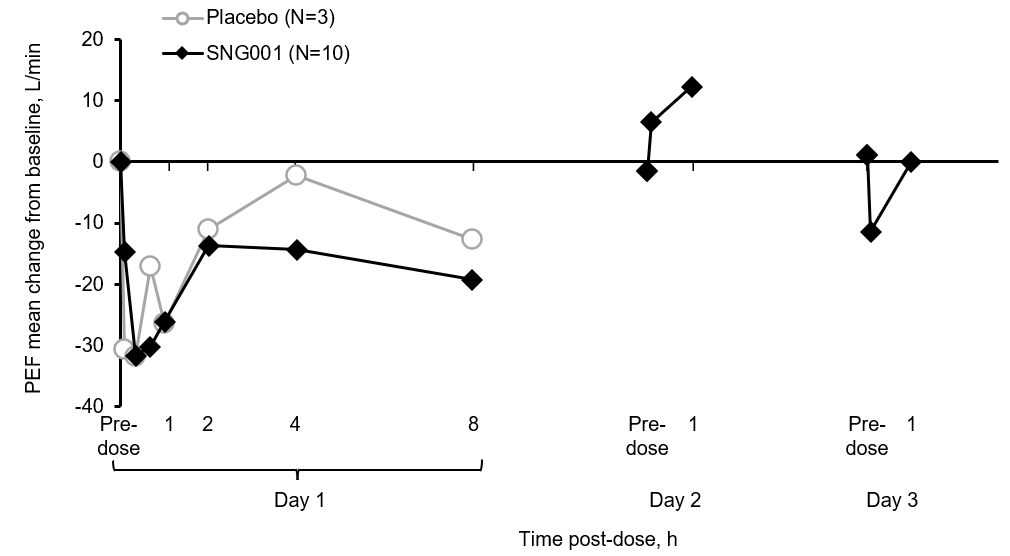


PEF, peak expiratory flow. Mean changes from baseline were not calculated for placebo on Days 2 or 3.

# Results – Part 2

Table S3. Part 2: CAT total score, assessed at clinic visits.

|  | **Group A (cold–like symptoms and/or deterioration in COPD symptoms)** | | **Group B (moderate COPD exacerbation)** | |
| --- | --- | --- | --- | --- |
|  | **Placebo (N=36)** | **SNG001 (N=38)** | **Placebo (N=16)** | **SNG001 (N=19)** |
| Baseline | 20.6 (8.21) | 21.0 (7.54) | 20.7 (5.35) | 28.0 (6.69) |
| Day 4, change from baseline | –2.7 (–5.1, –0.3) | –1.2 (–3.6, 1.1) | –0.9 (–4.8, 2.9) | –1.5 (–4.7, 1.7) |
| SNG001–placebo difference | 1.5 (–1.8, 4.8); p=0.369 | | –0.6 (–5.6, 4.4); p=0.812 | |
| Day 7, change from baseline | –1.9 (–4.6, 0.8) | –2.9 (–5.5, –0.3) | –2.1 (–6.4, 2.3) | –4.6 (–8.3, –1.0) |
| SNG001–placebo difference | –1.0 (–4.7, 2.7); p=0.588 | | –2.6 (–8.3, 3.1); p=0.366 | |
| Day 10, change from baseline | –3.6 (–6.3, –1.0) | –4.0 (–6.6, –1.5) | –2.1 (–6.4, 2.2) | –3.8 (–7.4, –0.2) |
| SNG001–placebo difference | –0.4 (–4.1, 3.3); p=0.825 | | –1.7 (–7.4, 3.9); p=0.538 | |
| Day 13, change from baseline | –4.6 (–7.3, –1.8) | –3.6 (–6.2, –0.9) | –2.8 (–7.2, 1.6) | –5.3 (–9.0, –1.6) |
| SNG001–placebo difference | 1.0 (–2.8, 4.8); p=0.592 | | –2.5 (–8.3, 3.3); p=0.396 | |
| Day 4–13, change from baseline | –3.2 (–5.4, –1.0) | –2.9 (–5.0, –0.8) | –2.0 (–5.5, 1.6) | –3.8 (–6.8, –0.9) |
| SNG001–placebo difference | 0.3 (–2.7, 3.3); p=0.857 | | –1.8 (–6.5, 2.8); p=0.424 | |

Baseline data are mean (SD); post-baseline data are least squares mean change from baseline and 95% confidence interval. CAT, COPD Assessment Test.

Table S4. Part 2: Proportion of patients whose symptoms returned to normal after a moderate exacerbation.

|  | **Group A (cold–like symptoms and/or deterioration in COPD symptoms)** | | **Group B (moderate COPD exacerbation)** | |
| --- | --- | --- | --- | --- |
|  | **Placebo (N=36)** | **SNG001 (N=38)** | **Placebo (N=16)** | **SNG001 (N=19)** |
| Day 4 | 11 (30.6%) | 8 (21.6%) | 0 | 3 (15.8%) |
| SNG001–placebo difference | 0.61 (0.21, 1.76); p=0.358 | | Not calculated | |
| Day 7 | 14 (38.9%) | 10 (27.8%) | 2 (13.3%) | 5 (26.3%) |
| SNG001–placebo difference | 0.60 (0.22, 1.61); p=0.309 | | 2.33 (0.38, 14.23); p=0.358 | |
| Day 10 | 18 (50.0%) | 15 (41.7%) | 4 (26.7%) | 5 (26.3%) |
| SNG001–placebo difference | 0.71 (0.28, 1.80); p=0.467 | | 0.93 (0.19, 4.47); p=0.931 | |
| Day 13 | 20 (55.6%) | 18 (48.6%) | 5 (31.3%) | 7 (38.9%) |
| SNG001–placebo difference | 0.75 (0.30, 1.89); p=0.540 | | 1.31 (0.31, 5.60); p= 0.713 | |

Treatment contrasts are presented as odds ratio and 95% confidence interval.

Table S5. Part 2: Post-bronchodilator spirometry endpoints, assessed at clinic visits.

|  | **Group A (cold–like symptoms and/or deterioration in COPD symptoms)** | | **Group B (moderate COPD exacerbation)** | |
| --- | --- | --- | --- | --- |
|  | **Placebo (N=36)** | **SNG001 (N=38)** | **Placebo (N=16)** | **SNG001 (N=19)** |
| **FEV_1_, L** |  |  |  |  |
| Baseline | 1.683 (0.5831) | 1.526 (0.5578) | 1.449 (0.3625) | 1.512 (0.5251) |
| Day 4, change from baseline | –0.003 (–0.058, 0.053) | –0.009 (–0.064, 0.045) | –0.052 (–0.137, 0.033) | –0.083 (–0.160, –0.007) |
| SNG001–placebo difference | –0.007 (–0.085, 0.071); p=0.867 | | –0.031 (–0.145, 0.082); p=0.585 | |
| Day 7, change from baseline | 0.007 (–0.049, 0.063) | –0.022 (–0.077, 0.033) | –0.014 (–0.099, 0.070) | –0.066 (–0.142, 0.011) |
| SNG001–placebo difference | –0.030 (–0.108, 0.049); p=0.458 | | –0.051 (–0.165, 0.062); p=0.374 | |
| Day 10, change from baseline | 0.015 (–0.045, 0.074) | –0.051 (–0.110, 0.008) | –0.030 (–0.120, 0.060) | –0.018 (–0.099, 0.063) |
| SNG001–placebo difference | –0.066 (–0.150, 0.018); p=0.121 | | 0.012 (–0.109, 0.133); p=0.844 | |
| Day 13, change from baseline | 0.022 (–0.041, 0.086) | –0.039 (–0.101, 0.022) | 0.045 (–0.049, 0.139) | –0.028 (–0.116, 0.060) |
| SNG001–placebo difference | –0.062 (–0.150, 0.027); p=0.171 | | –0.073 (–0.202, 0.055); p=0.261 | |
| Day 4–13, change from baseline | 0.010 (–0.040, 0.060) | –0.031 (–0.079, 0.018) | –0.013 (–0.088, 0.062) | –0.049 (–0.117, 0.019) |
| SNG001–placebo difference | –0.041 (–0.111, 0.029); p=0.248 | | –0.036 (–0.137, 0.065); p=0.481 | |
| **FVC, L** |  |  |  |  |
| Baseline | 3.328 (0.9161) | 3.059 (0.8223) | 3.036 (0.8096) | 2.911 (0.7847) |
| Day 4, change from baseline | 0.080 (–0.028, 0.188) | 0.028 (–0.078, 0.133) | –0.068 (–0.232, 0.097) | –0.022 (–0.170, 0.126) |
| SNG001–placebo difference | –0.052 (–0.204, 0.099); p=0.494 | | 0.046 (–0.175, 0.266); p=0.682 | |
| Day 7, change from baseline | 0.112 (–0.016, 0.239) | –0.018 (–0.143, 0.107) | 0.048 (–0.145, 0.240) | –0.012 (–0.186, 0.163) |
| SNG001–placebo difference | –0.130 (–0.309, 0.049); p=0.154 | | –0.059 (–0.318, 0.199); p=0.650 | |
| Day 10, change from baseline | 0.121 (–0.013, 0.254) | –0.099 (–0.230, 0.033) | 0.018 (–0.182, 0.219) | –0.023 (–0.205, 0.160) |
| SNG001–placebo difference | –0.219 (–0.407, –0.032); p=0.023 | | –0.041 (–0.311, 0.229); p=0.764 | |
| Day 13, change from baseline | 0.151 (0.015, 0.286) | –0.026 (–0.158, 0.105) | 0.166 (–0.034, 0.365) | 0.007 (–0.180, 0.195) |
| SNG001–placebo difference | –0.177 (–0.366, 0.012); p=0.066 | | –0.158 (–0.431, 0.115); p=0.253 | |
| Day 4–13, change from baseline | 0.116 (0.010, 0.222) | –0.029 (–0.132, 0.075) | 0.041 (–0.118, 0.200) | –0.012 (–0.158, 0.133) |
| SNG001–placebo difference | –0.145 (–0.293, 0.004); p=0.057 | | –0.053 (–0.267, 0.161); p=0.623 | |
| **FEV_1_/FVC, %** |  |  |  |  |
| Baseline | 50.57 (10.103) | 49.98 (11.559) | 49.03 (11.406) | 51.92 (11.548) |
| Day 4 | 49.17 (47.90, 50.43) | 50.08 (48.84, 51.32) | 50.18 (48.24, 52.11) | 48.25 (46.51, 49.99) |
| SNG001–placebo difference | 0.92 (–0.85, 2.69); p=0.307 | | –1.93 (–4.52, 0.67); p=0.144 | |
| Day 7 | 49.00 (47.65, 50.34) | 50.36 (49.03, 51.69) | 49.54 (47.49, 51.58) | 48.72 (46.88, 50.57) |
| SNG001–placebo difference | 1.36 (–0.52, 3.25); p=0.154 | | –0.81 (–3.56, 1.94); p=0.559 | |
| Day 10 | 49.32 (47.81, 50.83) | 50.73 (49.22, 52.24) | 49.57 (47.28, 51.87) | 49.58 (47.51, 51.66) |
| SNG001–placebo difference | 1.41 (–0.72, 3.54); p=0.193 | | 0.01 (–3.09, 3.10); p=0.996 | |
| Day 13 | 48.81 (47.26, 50.35) | 49.91 (48.39, 51.42) | 50.14 (47.83, 52.45) | 48.99 (46.81, 51.17) |
| SNG001–placebo difference | 1.10 (–1.06, 3.26); p=0.316 | | –1.15 (–4.33, 2.02); p=0.473 | |
| Day 4–13 | 49.07 (47.88, 50.26) | 50.27 (49.10, 51.44) | 49.86 (48.06, 51.65) | 48.89 (47.24, 50.53) |
| SNG001–placebo difference | 1.20 (–0.47, 2.87); p=0.158 | | –0.97 (–3.40, 1.46); p=0.430 | |

Baseline data are mean (SD); post-baseline data are least squares mean (change from baseline for FEV_1_ and FVC, and absolute value for FEV_1_/FVC %) and 95% confidence interval. FEV_1_, forced expiratory volume in 1 sec; FVC, forced vital capacity.

Figure S3. Part 2: Summary of baseline positive respiratory virus test results in Group A (cold-like symptoms and/or deterioration in COPD symptoms) and Group B (moderate COPD exacerbation).


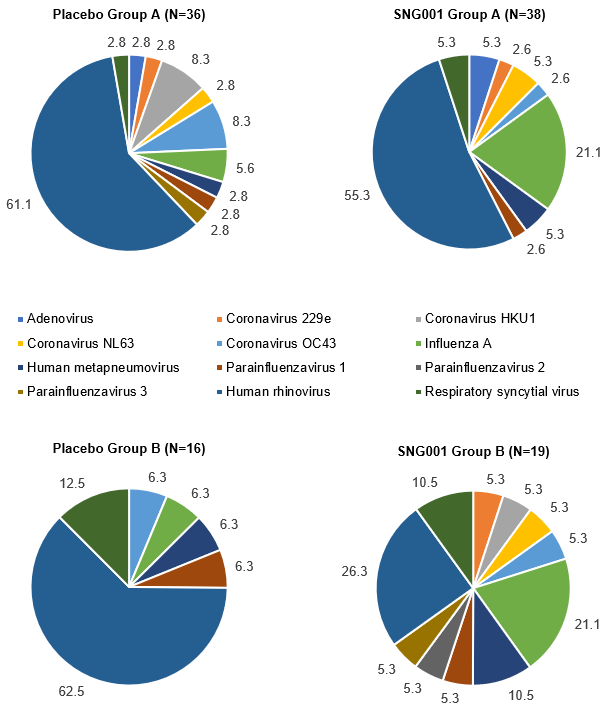


Note that patients could test positive for more than one respiratory virus.

Figure S4. Part 2: Serum antiviral interferon-stimulated gene products in Group A (cold-like symptoms and/or deterioration in COPD symptoms) and Group B (moderate COPD exacerbation).


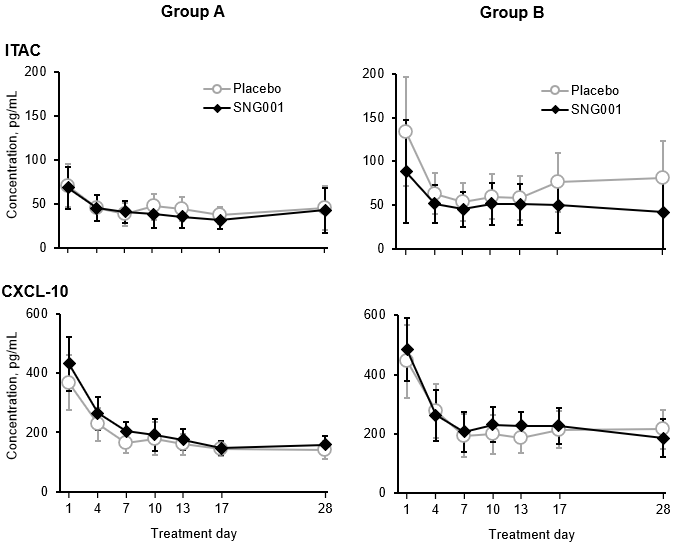


There were no statistically significant differences between treatments. Data are least squares mean and 95% confidence intervals. ITAC, interferon-inducible T-cell alpha chemoattractant, also known as CXCL11; CXCL10, C-X-C motif chemokine ligand 10. Data available at baseline from 30 and 31 patients receiving placebo and SNG001, respectively, in Group A, and 13 and 17 patients, respectively, in Group B.

Figure S5. Part 2: Serum inflammatory protein markers in Group A (cold-like symptoms and/or deterioration in COPD symptoms) and Group B (moderate COPD exacerbation).


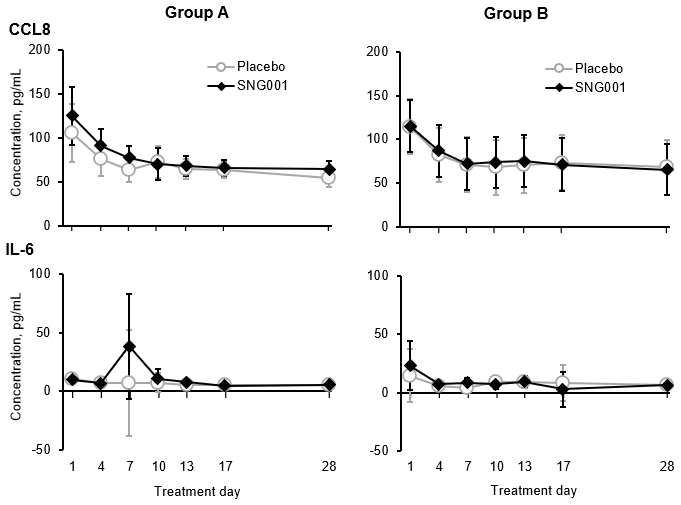


There were no statistically significant differences between treatments. Data are least squares mean and 95% confidence intervals. CCL8, chemokine (C-C motif) ligand 8; IL-6, interleukin 6. Data available at baseline from 30 and 31 patients receiving placebo and SNG001, respectively, in Group A, and 13 and 17 patients, respectively, in Group B.

Figure S6. Part 2: Serum CRP in Group A (cold-like symptoms).


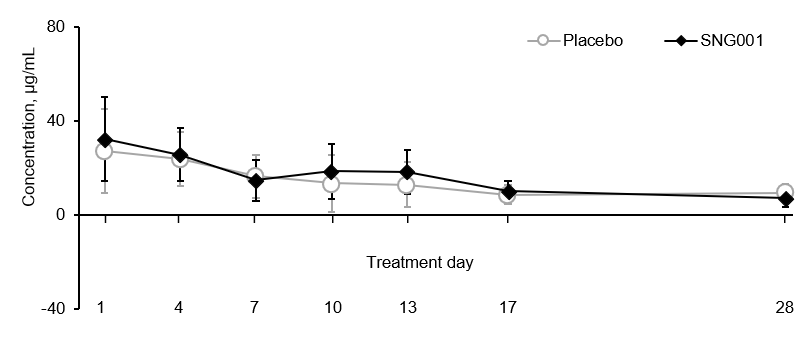


CRP, C-reactive protein. Data in the top figure are least squares mean and 95% confidence intervals, with data available at baseline from 30 and 31 patients receiving placebo and SNG001, respectively.

Figure S7. Part 2: Proportion of patients with purulent sputum in Group A (cold-like symptoms.


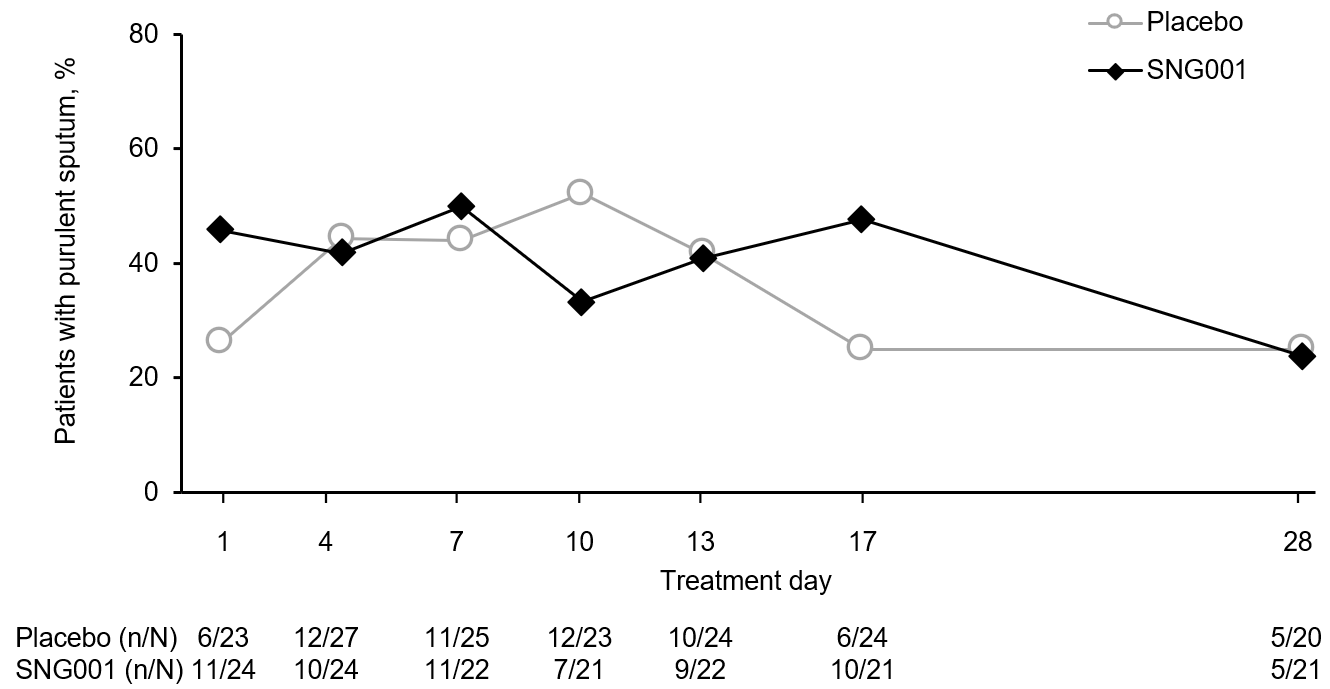


There were no statistically significant differences between treatments. Sputum was graded on a colour scale from 1 to 5 where 1 indicated that antibiotics would not be usually required and 5 indicated that antibiotics may be required, with Grades of 3 to 5 reflecting ‘purulent’ sputum. Percentages are calculated as the number of patients with purulent sputum (n) divided by the number with non-missing data (N).
